# NSUN2-mediated RNA 5-methylcytosine promotes esophageal squamous cell carcinoma progression via LIN28B-dependent GRB2 mRNA stabilization

**DOI:** 10.1038/s41388-021-01978-0

**Published:** 2021-08-03

**Authors:** Jiachun Su, Guandi Wu, Ying Ye, Jialiang Zhang, Lingxing Zeng, Xudong Huang, Yanfen Zheng, Ruihong Bai, Lisha Zhuang, Mei Li, Ling Pan, Junge Deng, Rui Li, Shuang Deng, Shaoping Zhang, Zhixiang Zuo, Zexian Liu, Junzhong Lin, Dongxin Lin, Jian Zheng

**Affiliations:** 1 State Key Laboratory of Oncology in South China and Collaborative Innovation Center for Cancer Medicine, Sun Yat-sen University Cancer Center, Guangzhou, China; 2 Department of Pathology, Sun Yat-sen University Cancer Center, Guangzhou, China; 3 Department of Surgery, Sun Yat-sen University Cancer Center, Guangzhou, China; 4 Department of Etiology and Carcinogenesis, National Cancer Center/National Clinical Research Center/Cancer Hospital, Chinese Academy of Medical Sciences and Peking Union Medical College, Beijing, China; 5 Jiangsu Key Lab of Cancer Biomarkers, Prevention and Treatment, Collaborative Innovation Center for Cancer Medicine, Nanjing Medical University, Nanjing, China

**Keywords:** Oesophageal cancer, Epigenetics, RNA metabolism

## Abstract

5-Methylcytosine (m^5^C) is a posttranscriptional RNA modification participating in many critical bioprocesses, but its functions in human cancer remain unclear. Here, by detecting the transcriptome-wide m^5^C profiling in esophageal squamous cell carcinoma (ESCC), we showed increased m^5^C methylation in ESCC tumors due to the overexpressed m^5^C methyltransferase NSUN2. Aberrant expression of *NSUN2* was positively regulated by E2F Transcription Factor 1 (E2F1). High *NSUN2* levels predicted poor survival of ESCC patients. Moreover, silencing *NSUN2* suppressed ESCC tumorigenesis and progression in *Nsun2* knockout mouse models. Mechanistically, NSUN2 induced m^5^C modification of growth factor receptor-bound protein 2 (*GRB2*) and stabilized its mRNA, which was mediated by a novel m^5^C mediator, protein lin-28 homolog B (LIN28B). Elevated *GRB2* levels increased the activation of PI3K/AKT and ERK/MAPK signalling. These results demonstrate that NSUN2 enhances the initiation and progression of ESCC via m^5^C-LIN28B dependent stabilization of *GRB2* transcript, providing a promising epitranscriptomic-targeted therapeutic strategy for ESCC.

## Introduction

Esophageal squamous cell carcinoma (ESCC) is one of the most malignant cancers with only ~19% of 5-year survival [1, 2]. Most ESCC patients eventually die of cancer progression due to lack of effective treatment modalities [3]. Therefore, further elucidation of the comprehensive molecular mechanisms underlying ESCC is urgently needed to develop more effective diagnostic and therapeutic interventions for ESCC.

Recent discoveries have demonstrated that aberrations in epigenetic regulation, such as RNA methylation, are crucial hallmarks of tumor initiation, progression, and recurrence [4]. 5-Methylcytosine (m^5^C) is one of the most well-known and conserved RNA modifications extensively occurring on various types of eukaryotic RNA, including rRNA, lncRNA, tRNA, and mRNA [5–10]. To date, the known m^5^C methyltransferases include the NOP2/Sun RNA methyltransferase family member 1−7 (NSUN1−7) and the DNA methyltransferase 2 [11]. Aly/REF export factor (ALYREF) and Y-box binding protein 1 (YBX1) are two m^5^C readers respectively mediating RNA nuclear export and enhancing RNA stability [9, 12, 13]. Accumulating evidence confirms that m^5^C modification regulates multiple RNA metabolic and biological processes, such as RNA stability [12, 13], RNA export [9], RNA translation [14, 15], and RNA processing [16, 17]. Recently, with the advance in detecting and mapping techniques, distribution profiles of m^5^C sites on mRNAs have been discovered, suggesting that m^5^C sites are distributed in 5′ untranslated regions (5′UTR), coding sequences (CDS) and 3′ untranslated regions (3′UTR) of mRNAs and are especially prominent near translation start sites [8, 9]. NSUN2 and NSUN6 are two major methyltransferase catalyzing m^5^C modification of mammalian mRNAs [7, 9, 11, 18] and NSUN2 is currently the best-studied one. Emerging evidence has shown that NSUN2-mediated RNA m^5^C methylation plays a critical role in cell proliferation, development, and differentiation [16,19–21]. Mutation or aberrant expression of NSUN2 is involved in various diseases, such as cancer and developmental disorders [12, 21−23]. However, very little is known about the precise regulatory mechanism of NSUN2-mediated mRNA m^5^C modification in human diseases, especially human cancer.

In this study, we indicate an oncogenic role of NSUN2-mediated RNA m^5^C modification in human ESCC. Specifically, E2F Transcription Factor 1 (E2F1) binds to the promoter of *NSUN2* and enhances its expression, which significantly increases m^5^C formation in growth factor receptor-bound protein 2 (*GRB2*). An RNA-binding protein lin-28 homolog B (LIN28B) acts as an m^5^C mediator preferentially binding to the m^5^C-modified *GRB2* RNA and enhancing its stability. Upregulation of GRB2 evokes the oncogenic PI3K/AKT and ERK/MAPK signaling. We propose that the NSUN2-m^5^C-GRB2-PI3K/AKT and ERK/MAPK signaling axes promote the initiation and the progression of ESCC.

## Results

### Aberrant upregulation of *NSUN2* plays an oncogenic role in ESCC

**Fig. 1:**
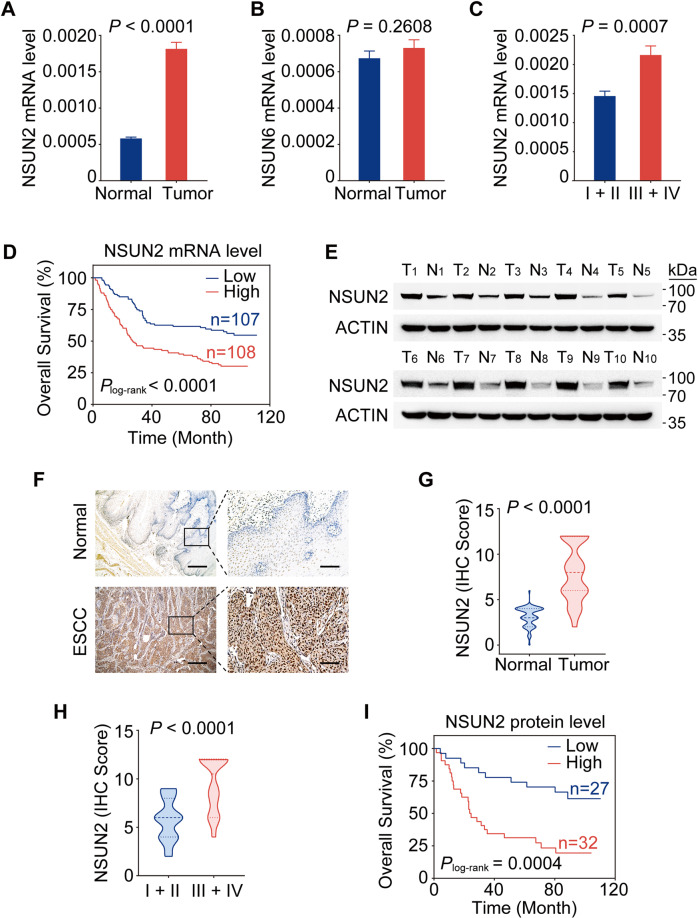
Elevated *NSUN2* expression correlates with poor clinical outcomes in patients with ESCC. **A**, **B***NSUN2* RNA levels were significantly higher in ESCC tumors than in paired normal tissues (*n* = 215; SYSUCC cohort) (**A**), but *NSUN6* showed no obvious difference (**B**). **C** Increased *NSUN2* RNA levels were observed in stage III/IV ESCC tumors (*n* = 109) than in stage I/II tumors (*n* = 106). **D** Kaplan–Meier estimates of survival time of patients in the SYSUCC cohort by different *NSUN2* RNA levels in tumors. The median survival time for patients with high *NSUN2* (≥median) was 26.9 months, significantly shorter than 87.7 months in patients with low *NSUN2* (<median), with the adjusted HRs (95% CI) for death of high *NSUN2* level being 1.94 (1.32–2.85). HR hazard ratio, CI confidence interval. **E** Western blotting analysis showing higher levels of NSUN2 protein in ESCC tumors than in paired normal tissues (*n* = 10). T tumor tissues, N paired normal tissues. **F** Representative immunohistochemical staining (IHC) images of NSUN2 protein levels in ESCC tumors and in paired normal tissues. Scale bar, 500 μm (left) and 100 μm (right). **G**, **H** NSUN2 protein levels were significantly higher in ESCC tumors than in paired normal tissues (**G**; *n* = 59; SYSUCC cohort), and in stage III/IV tumors (*n* = 34) than in stage I/II tumors (*n* = 25) (**H**). **I** Kaplan–Meier estimates of survival time of patients by different NSUN2 protein levels in tumors. The median survival time for patients with high NSUN2 (IHC score > 6; 24.2 months) was significantly shorter than those with low NSUN2 (IHC score ≤ 6; 88.4 months), with the adjusted HRs (95% CI) for death of high NSUN2 level being 3.52 (1.46–8.49). Data are represented as mean ± SEM in (**A**–**C**) and violin plots in (**G**) and (**H**). The centerlines of the violin plots represent median, while the upper and lower hinges indicate 25th and 75th percentiles, respectively. *P* values were calculated by two-sided paired Wilcoxon signed-rank test in (**A**), (**B**) and (**G**), and two-sided Mann-Whitney test in (**C**) and (**H**). Two-sided log-rank test was used in (**D**) and (**I**).

To determine the role of mRNA m^5^C modification in ESCC, we evaluated the expression levels of two major mRNA m^5^C methyltransferases NSUN2 and NSUN6 in an ESCC cohort (*n* = 215; Supplementary Table 1) from Sun Yat-sen University Cancer Center (SYSUCC) using qRT-PCR. We found aberrantly higher levels of *NSUN2* RNA in tumors than in adjacent normal tissues (Fig. 1A). However, no obvious difference of *NSUN6* RNA was observed (Fig. 1B). These findings were further verified in a public microarray dataset (GSE53625) consisting of 179 paired ESCC samples (Supplementary Fig. 1A, B). Furthermore, higher *NSUN2* RNA was significantly associated with advanced ESCC tumor stage (Supplementary Table 2). Patients with stages III/IV ESCCs showed higher *NSUN2* levels than those with stages I/II ESCCs (Fig. 1C). Kaplan–Meier analysis revealed that patients with high *NSUN2* RNA levels had shorter overall survival times (OS) than those with low levels (Fig. 1D), with an adjusted HR of death for high level being 1.94 (95% CI, 1.32–2.85) (Supplementary Table 3).

We further assessed the protein levels of *NSUN2* in ESCC and observed that NSUN2 protein was expressed at significantly higher levels in ESCC tumors than in paired normal tissues by western blotting (*n* = 10; Fig. 1E) and by immunohistochemical staining (IHC) (*n* = 59; Fig. 1F, G; Supplementary Table 4). Consistently, NSUN2 protein levels were also significantly correlated with tumor stages (Supplementary Table 5). Higher NSUN2 protein levels were found in stages III/IV ESCCs than in stages I/II ESCCs (Fig. 1H) and were correlated with worse prognosis (Fig. 1I; Supplementary Table 6). These results suggest that *NSUN2* is frequently upregulated in ESCC and might serve as a prognostic indicator for ESCC patients.

### *NSUN2* expression is positively regulated by E2F1

**Fig. 2:**
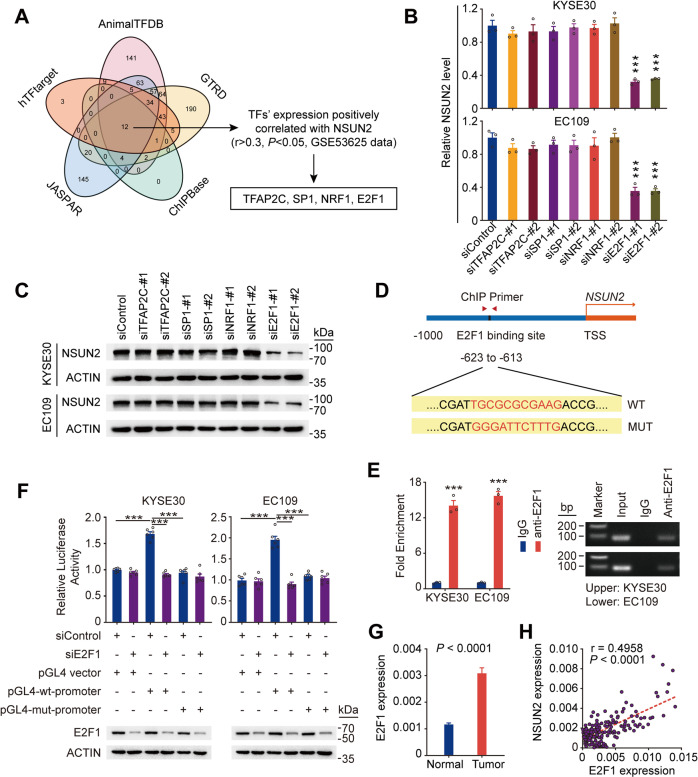
Transcription factor E2F1 positively regulates *NSUN2* expression in ESCC. **A** In silico analysis of potential transcription factors in *NSUN2* promoter region. **B**, **C** Relative *NSUN2* RNA (**B**) and protein (**C**) levels in ESCC cells with or without knockdown of each of the four transcription factors indicated in (**A**). **D** Schema of the putative E2F1 binding site in *NSUN2* promoter and the primers used for chromatin immunoprecipitation (ChIP) analysis. Highlighted are the consensus and mutant sequences for E2F1 binding. **E** ChIP-qPCR analysis of cells with anti-E2F1 antibody or IgG control. Left panel shows qPCR results and the right panel shows the images of agarose gel electrophoresis of the qPCR products. **F** Luciferase reporter assays in cells co-transfected with the indicated plasmids or siRNA for 48 h (upper panel). Knockdown efficiency of *E2F1* was shown in the lower panel. pGL4-wt-promoter, pGL4-*NSUN2*-wt-promoter; pGL4-mut-promoter, pGL4-*NSUN2*-mut-promoter. **G** *E2F1* RNA levels were significantly higher in ESCC tumors than in paired normal tissues (*n* = 215, SYSUCC cohort). **H** Spearman’s correlations between RNA levels of *NSUN2* and *E2F1* in ESCC tumors (*n* = 215). The center red line is the regression line. r, correlation coefficient. Data are shown as mean ± SEM in (**B**), (**E**), (**F**) and (**G**). All data are from at least three independent experiments. ACTIN was used as a control in (**C**) and (**F**). *P* values were calculated by two-sided Student’s *t* test (*P < 0.05, **P < 0.01 and ***P < 0.001) in (**B**), (**E**) and (**F**), and two-sided paired Wilcoxon signed-rank test in (**G**).

To explore why *NSUN2* is overexpressed in ESCC, we looked at genomic alterations including CNV and methylation status of *NSUN2* in ESCC tissues derived from different datasets but the results were negative (Supplementary Fig. 2A, B). We then performed in silico analysis using five publicly available databases (ChIPBase, GTRD, AnimalTFDB, JASPAR and hTFtarget) to search for cis-element(s) in the *NSUN2* promoter region (from −1000 bp to the transcription start site) and also looked at a public microarray dataset of 179 paired ESCC (GSE53625) to search for the transcription factors (TFs) positively correlated with *NSUN2* levels (*r* > 0.3, *P* < 0.05). By overlapping the results from these approaches, we identified four potential TFs for *NSUN2* (Fig. 2A; Supplementary Fig. 2C). Among the four TFs, only silencing E2F1 significantly downregulated *NSUN2* at both RNA and protein levels in ESCC cells (Fig. 2B, C; Supplementary Fig. 2D). However, no significant alteration of *NSUN6* level was observed when *E2F1* was silenced in ESCC cells (Supplementary Fig. 2E). In silico analysis indicated that cis-element for E2F1 might be located between −623 and −613 bp upstream of the *NSUN2* transcriptional start site (Fig. 2D), which was verified by chromatin immunoprecipitation (ChIP) assays (Fig. 2E). Moreover, luciferase reporter assays showed a significant increased transcriptional activity of *NSUN2* wild-type promoter as compared to the empty vector or vector with putative E2F1 binding motif mutant *NSUN2* promoter (Fig. 2F). This upregulation of luciferase expression was abrogated when *E2F1* was silenced (Fig. 2F). Further analysis of the GSE53625 dataset and our in-house 215 paired ESCC cohort showed that *E2F1* levels were significantly upregulated in ESCC tumors (Fig. 2G; Supplementary Fig. 2F). Consistently, *NSUN2* levels were also positively correlated with *E2F1* levels in our 215 paired ESCC samples (Fig. 2H). All these data suggest an E2F1-dependent positive regulation on *NSUN2* expression in ESCC.

### NSUN2 enhances tumorigenesis and progression of ESCC

**Fig. 3:**
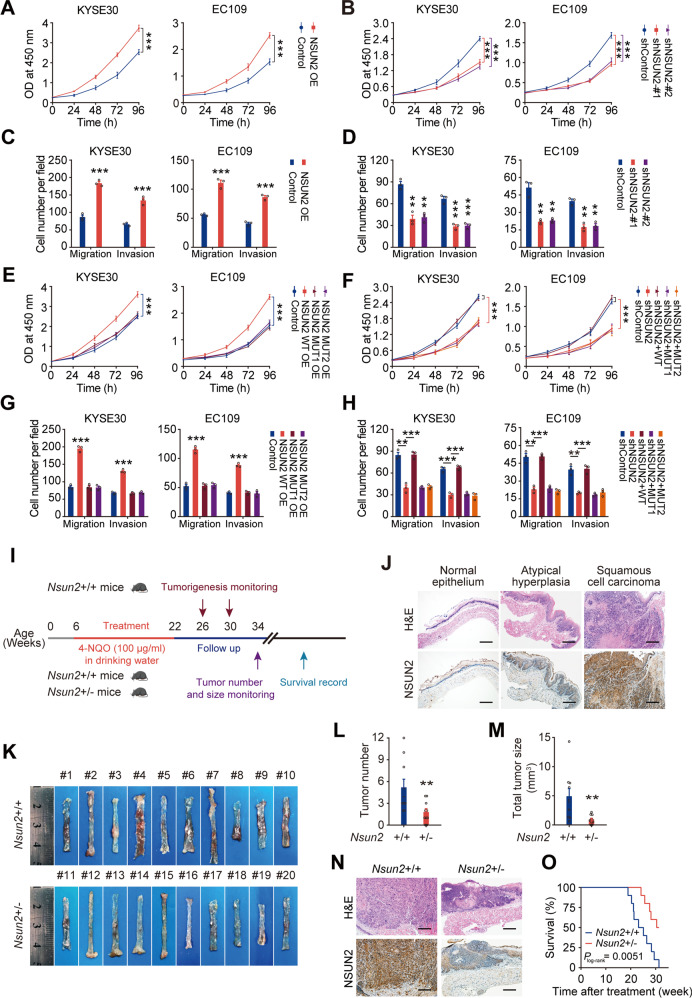
NSUN2 promotes malignant phenotypes of ESCC cells and enhances the tumorigenesis and development of *Nsun2* knockout mice. **A**−**D** Effects of *NSUN2* overexpression or knockdown on abilities of ESCC cell proliferation (**A**, **B**), migration and invasion (**C**, **D**). **E**, **G** Wild-type but not mutant *NSUN2* enhanced proliferation (**E**), migration and invasion abilities (**G**) of ESCC cells. **F**, **H** Wild-type but not mutant *NSUN2* reversed the decreased abilities of proliferation (**F**), migration and invasion (**H**) in *NSUN2*-depleted ESCC cells. WT, wild-type *NSUN2* plasmids; MUT1, *NSUN2* plasmid with a point mutation at catalytic site (C321A); MUT2, *NSUN2* plasmid with point mutations at both catalytic site (C321A) and releasing site (C271A). All three plasmids were insensitive to shNSUN2 plasmid. **I** Schematic diagram of the timeline of establishing the 4-NQO-induced mouse model of ESCC. Colored arrows indicate the time when different events occurred. **J** Pathological features (upper panel) or NSUN2 levels (lower panel) of normal esophagus or esophagus tissues after 4-NQO withdrawal 4 or 8 weeks from *Nsun2*+/+ mice, as estimated by hematoxylin and eosin (H&E) staining or immunohistochemical staining (IHC). **K**−**M** Morphological images (**K**), tumor number (**L**) or tumor size (**M**) of esophagus collected from *Nsun2*+/+ mice (*n* = 10) and their *Nsun2*+/− (*n* = 10) littermate after 4-NQO withdraw for 12 weeks. **N** Pathological features (upper panel) or NSUN2 levels (lower panel) of esophagus collected from *Nsun2*+/+ mice and their *Nsun2*+/− littermate after 4-NQO withdraw for 12 weeks, as estimated by H&E or IHC. **O** Kaplan–Meier analysis showing significantly longer survival times in *Nsun2*+/− mice (*n* = 10) than in *Nsun2*+/+ mice (*n* = 10). Results of (**A**−**H**) are from at least three independent experiments. Data in (**A**−**H**) and (**L**, **M**) are displayed as mean ± SEM. Scale bars are 200 µm in (**J**) and (**N**). *P* values were calculated by two-sided Student’s *t* test in (**A, H**), two-sided Mann–Whitney test in (**L, M**), and two-sided log-rank test in (**O**). *P < 0.05, **P < 0.01 and ***P < 0.001.

We then investigate the role of NSUN2 by changing its expression in ESCC cells. Overexpressing *NSUN2* substantially promoted cell proliferation, migration and invasion, while silencing *NSUN2* showed opposite effects (Fig. 3A−D; Supplementary Fig. 3A, B, E, F). However, overexpressing *NSUN2* catalytic mutants (MUT1 or MUT2) [9, 12] had no obvious effects on malignant phenotypes of ESCC cells (Fig. 3E, G; Supplementary Fig. 3C, G). Moreover, overexpressing wild-type but not mutant *NSUN2* substantially reversed the inhibitory effects of *NSUN2* knockdown on malignant phenotypes of ESCC cells (Fig. 3F, H; Supplementary Fig. 3D, H). These in-vitro results suggest an oncogenic role of NSUN2 in ESCC cells.

We next generated *Nsun2* knockout mice (*Nsun2*+/−, Supplementary Fig. 4A) to determine the oncogenic role of *Nsun2* in vivo. A 14 bp shift due to the deletion mutation located on one allele of *Nsun2* exon 4 was observed in the *Nsun2*+/− mice by Sanger sequencing (Supplementary Fig. 4B). Relative to those of *Nsun2*+/+ littermates, esophageal extracts of *Nsun2*+/− mice displayed significant reduction in NSUN2 protein levels (Supplementary Fig. 4C). We then used the *Nsun2*+/− mice and their wild-type (*Nsun2*+/+) littermates to induce ESCC by treating them with chemical carcinogen 4-nitroquilonine *N*-oxide (4-NQO) (Fig. 3I). We observed esophageal atypical hyperplasia lesions or squamous cell carcinoma in *Nsun2*+/+ mice after 4-NQO withdrawal for 4 or 8 weeks. IHC assays showed sequential upregulation of NSUN2 protein in normal esophageal epithelium, atypical hyperplasia lesions and ESCC tumor tissues (Fig. 3J). As expected, all (10/10) *Nsun2*+/+ mice developed esophageal masses after 4-NQO withdrawal for 12 weeks, but not all (8/10) *Nsun2*+/− mice at this timepoint (Fig. 3K). Both tumor number and tumor size of *Nsun2*+/− mice were smaller than those of *Nsun2*+/+ mice (Fig. 3L, M). Histopathological analysis showed advanced esophageal tumor stages of *Nsun2*+/+ mice than those of *Nsun2*+/− mice (Fig. 3N; Supplementary Fig. 4D). Consistently, *Nsun2*+/+ mice had worse prognosis than *Nsun2*+/− mice (Fig. 3O). These findings suggest that NSUN2 plays a critical role in the tumorigenicity and progression of ESCC.

### NSUN2-mediated m^5^C hypermethylation activates PI3K/AKT and ERK/MAPK signaling in ESCC

We next explore whether the oncogenic role of NSUN2 is m^5^C-dependent in ESCC. We performed RNA bisulfite sequencing (RNA-BisSeq) [9] on poly(A)-enriched RNAs purified from seven paired ESCC samples (Supplementary Table 7) to elucidate the transcriptomic m^5^C profile of ESCC. Conversion rates (C to T) of the methylation conversion control *Dhfr* were all >99%. We identified 115,187 m^5^C sites in 8263 transcripts. More than 90% of these m^5^Cs occurred in mRNAs (Supplementary Fig. 5A) and were enriched in 5’UTR, CDS and 3′UTR (Supplementary Fig. 5B). Moreover, the identified m^5^Cs were particularly accumulated in regions immediately downstream of translation initiation sites (Supplementary Fig. 5C) and were enriched in the CG-rich environments (Supplementary Fig. 5D).

**Fig. 4:**
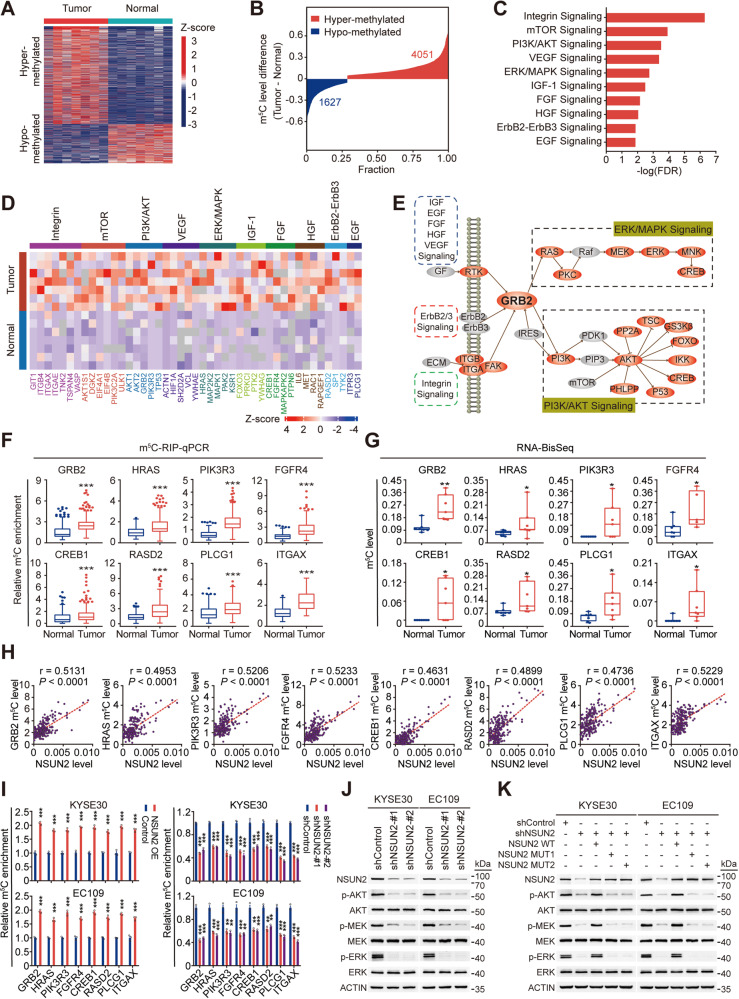
NSUN2 stimulates m^5^C hypermethylation in ESCC and activates PI3K/AKT and ERK/MAPK signaling. **A**, **B** Heatmap (**A**, left) and distribution (**B**) of the differential methylation levels of m^5^C sites between ESCC tumors and paired normal tissues (*n* = 7). **C** Canonical pathway analysis of genes with m^5^C hypermethylated transcripts (*n* = 1362) in ESCC tumors than in paired normal tissues (*n* = 7) using IPA software. **D** Heatmap (upper) showing m^5^C hypermethylation of 45 representative genes involved in ten canonical cancer-related pathways in ESCC tumors. **E** Schematic diagram of genes involved in these cancer-related pathways. Genes with m^5^C hypermethylated transcripts in tumors are highlighted in red. **F**, **G** Substantially hypermethylated m^5^C of representative transcripts involved in these cancer-related pathways in ESCC tumors than in paired normal tissues by m^5^C-RIP-qPCR (**F**, *n* = 215) or RNA-BisSeq (**G**, *n* = 7). **H** Spearman’s correlation analysis between *NSUN2* RNA levels and m^5^C levels of transcripts mentioned in (**F**) in ESCC tumors (*n* = 215). **I** Relative m^5^C levels of transcripts mentioned in (**F**) were increased or decreased in ESCC cells with *NSUN2* overexpression or knockdown. **J**, **K** Western blotting analysis showing that *NSUN2* depletion substantially suppressed AKT, MEK and ERK phosphorylation and activation in ESCC cells (**J**), while wild-type but not mutant *NSUN2* reversed the decrease of AKT, MEK and ERK phosphorylation and activation caused by *NSUN2* knockdown (**K**). Heatmaps in (**A**, right) and (**D**, lower) showing the *z*-score of m^5^C levels. Colors from blue to red indicate low to high. Data in (**F**, **G**) are displayed in boxplots. Data in (**I**) are shown as mean ± SEM from three independent experiments. The relative m^5^C levels in target transcripts in (**F**) and (**I**) were evaluated with input normalization. ACTIN was used as a control in (**J**, **K**). *P* values were calculated by two-sided paired Wilcoxon signed-rank test in (**F**), two-sided Mann–Whitney test in (**G**) and two-sided Student’s *t* test in (**I**) (*P < 0.05, **P < 0.01 and ***P < 0.001).

Among the 115,187 m^5^Cs, 4051 sites within 1362 transcripts were hypermethylated while 1627 sites within 626 transcripts were hypomethylated in tumors compared with those in normal tissues (Fig. 4A, B; Supplementary Data 1), indicating that m^5^C hypermethylation is a frequent event in ESCC. Furthermore, we found that RNA levels of *NSUN2* but not *NSUN6* were significantly upregulated in tumors from the seven paired ESCC samples (Supplementary Fig. 5E), suggesting that NSUN2 may be the main methyltransferase mediating the formation of aberrant mRNA m^5^C hypermethylation in ESCC. Then, pathway enrichment analysis using IPA software showed that the m^5^C-hypermethylated transcripts in tumors were mainly enriched in cancer-related pathways, such as PI3K/AKT, ERK/MAPK and cell-surface activated receptor-related pathways (Fig. 4C). Indeed, many oncogenes involved in these pathways exhibited m^5^C-hypermethylated in tumors (Fig. 4D, E). We randomly selected several transcripts involved in these pathways and verified their upregulated m^5^C levels in tumors (*n* = 215; Fig. 4F; Supplementary Table 1) using m^5^C-RIP-qPCR, which was consistent with our RNA-BisSeq results (Fig. 4G). Moreover, m^5^C levels of these transcripts were positively correlated with *NSUN2* RNA levels in ESCC tumors (*n* = 215; Fig. 4H). Consistently, silencing *NSUN2* decreased m^5^C levels of these transcripts in ESCC cells, while overexpressing *NSUN2* produced opposite effects (Fig. 4I). In *NSUN2*-depleted cells, wild-type but not mutant *NSUN2* restored m^5^C levels of these transcripts (Supplementary Fig. 5F).

Previous studies have reported that PI3K/AKT and ERK/MAPK pathways are two major downstream pathways of multiple cell-surface activated receptor-related (VEGF, integrin, etc.) pathways [24, 25], and many transcripts in these pathways were hypermethylated in our results (Fig. 4E). We thus hypothesized that NSUN2-mediated m^5^C hypermethylation might promote ESCC tumor progression mainly by regulating PI3K/AKT and ERK/MAPK signaling. As expected, *NSUN2* knockdown suppressed the activation of PI3K/AKT and ERK/MAPK pathways (Fig. 4J). This suppression could be reversed by overexpression of wild-type but not mutant *NSUN2* (Fig. 4K). These results indicate that NSUN2-mediated m^5^C hypermethylation may trigger PI3K/AKT and ERK/MAPK pathways to promote ESCC malignancy.

### GRB2 is a critical target via which NSUN2 stimulates PI3K/AKT and ERK/MAPK signaling

**Fig. 5:**
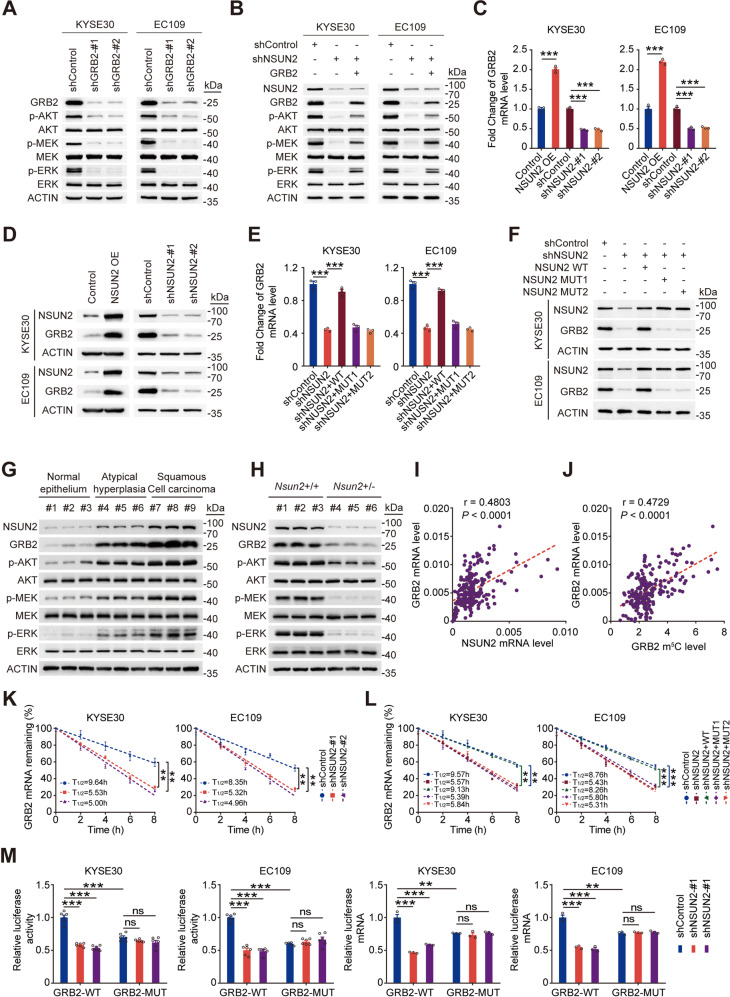
NSUN2 stimulates oncogenic PI3K/AKT and ERK/MAPK signaling by enhancing *GRB2* mRNA stability. **A** Western blotting analysis showing substantial inhibition of AKT, MEK and ERK phosphorylation in *GRB2*-depleted ESCC cells. **B** Western blotting analysis showing that overexpression of *GRB2* partially rescued the inhibitory effect of *NSUN2* knockdown on phosphorylation of AKT, MEK and ERK in ESCC cells. **C**, **D** Significant increase or decrease in *GRB2* mRNA (**C**) and protein (**D**) levels in *NSUN2* overexpression or knockdown ESCC cells. **E**, **F** Wild-type but not mutant *NSUN2* reversed the decrease of *GRB2* mRNA (**E**) and protein (**F**) levels caused by *NSUN2* depletion. **G** Expression levels of *GRB2*, p-AKT, p-MEK or p-ERK in esophagus tissues of different pathological stage from *Nsun2*+/+ mice. **H** Expression levels of *GRB2*, p-AKT, p-MEK or p-ERK in esophagus tissues from *Nsun2*+/+ mice and their *Nsun2*+/− littermate 12 weeks after 4-NQO withdraw. **I**, **J** Spearman’s correlation analysis between *GRB2* and *NSUN2* mRNA levels (**I**) or between *GRB2* mRNA and m^5^C levels (**J**) in ESCC tumors (*n* = 215) from the SYSUCC cohort. **K** RNA stability assays displaying reduced *GRB2* mRNA half-life in *NSUN2* knockdown ESCC cells compared with the control cells by qRT-PCR at the indicated time points after treatment with 5 µg/mL actinomycin D. **L** Wild-type but not mutant *NSUN2* reversed the decrease of *GRB2* mRNA half-life induced by *NSUN2* silencing. **M** Relative luciferase activity or mRNA levels of luciferase reporter gene with wild-type *GRB2*-m^5^C site (GRB2-WT) or mutant m^5^C site (GRB2-MUT) in control or *NSUN2* knockdown ESCC cells. Data are displayed as mean ± SEM in (**C**), (**E**), (**K**–**M**). All data are from at least three independent experiments. Two-sided Student’s *t* tests were used in (**C**), (**E**), (**K**–**M**) (*P < 0.05, **P < 0.01 and ***P < 0.001. ns not significant). ACTIN served as a control in (**A**), (**B**), (**D**) and (**F**–**H**).

To identify downstream effectors involved in NSUN2-mediated activation of PI3K/AKT and ERK/MAPK signaling, we assessed potential targets with hypermethylated-m^5^Cs in ESCC tumors. We focused on the adaptor protein GRB2 for the following reasons. Firstly, GRB2 plays a central role in signal transduction between cell-surface receptors and PI3K/AKT and ERK/MAPK signalling, leading to simultaneous activation of both PI3K/AKT and ERK/MAPK pathways [26, 27] (Fig. 4E). Secondly, results above had showed that NSUN2 positively regulated m^5^C levels of *GRB2* RNA via its m^5^C catalytic activity (Fig. 4I; Supplementary Fig. 5F), indicating that *GRB2* was a substrate of NSUN2. Further experiments showed that *GRB2* silencing substantially inhibited PI3K/AKT and ERK/MAPK signaling in ESCC cells (Fig. 5A). Overexpressing *GRB2* partly restored the activation of PI3K/AKT and ERK/MAPK pathways in *NSUN2*-depleted cells (Fig. 5B), indicating that *GRB2* is a key target of NSUN2 to activate both pathways.

We then explored the effect of NSUN2-mediated m^5^C formation on *GRB2*. *NSUN2* knockdown significantly reduced the *GRB2* RNA and protein levels in ESCC cells, while *NSUN2* overexpression exhibited opposite results (Fig. 5C, D). Moreover, overexpressing wild-type but not mutant *NSUN2* recovered the *GRB2* RNA (Fig. 5E) and protein (Fig. 5F) levels in *NSUN2*-knockdown cells. Consistently, we found both GRB2 levels and the activation levels of PI3K/AKT and ERK/MAPK pathways gradually increased in normal esophageal epithelium, atypical hyperplasia lesions and ESCC tumors in the 4-NQO-induced mouse model of ESCC (Fig. 5G). Higher levels of GRB2 and the activation levels of both pathways were also observed in esophageal tissues of *Nsun2*+/+ mice than in those of *Nsun2*+/− mice (Fig. 5H). Moreover, positive correlations were found between *GRB2* RNA levels and *NSUN2* RNA levels (Fig. 5I) or *GRB2* m^5^C levels (Fig. 5J) in ESCCs (*n* = 215; Supplementary Table 1). Since the m^5^C site of *GRB2* is in its 3′UTR, it is possible that NSUN2-mediated m^5^C modification maintains *GRB2* expression by enhancing its mRNA stability. We treated ESCC cells with actinomycin D and found that *NSUN2* silencing significantly reduced half-life of *GRB2* RNA (Fig. 5K). This reduction could be restored by overexpressing wild-type but not mutant *NSUN2* (Fig. 5L). Luciferase reporter assays showed substantial decreased luciferase expression of vector with wild-type *GRB2* 3′UTR (GRB2-WT), but not of that with m^5^C site mutant *GRB2* 3′UTR (GRB2-MUT) in *NSUN2*-depleted cells (Fig. 5M). These results suggest a central role of GRB2 in the NSUN2-enhanced activation of PI3K/AKT and ERK/MAPK signaling.

### LIN28B recognizes m^5^C modification of *GRB2* and stabilizes *GRB2* mRNA

**Fig. 6:**
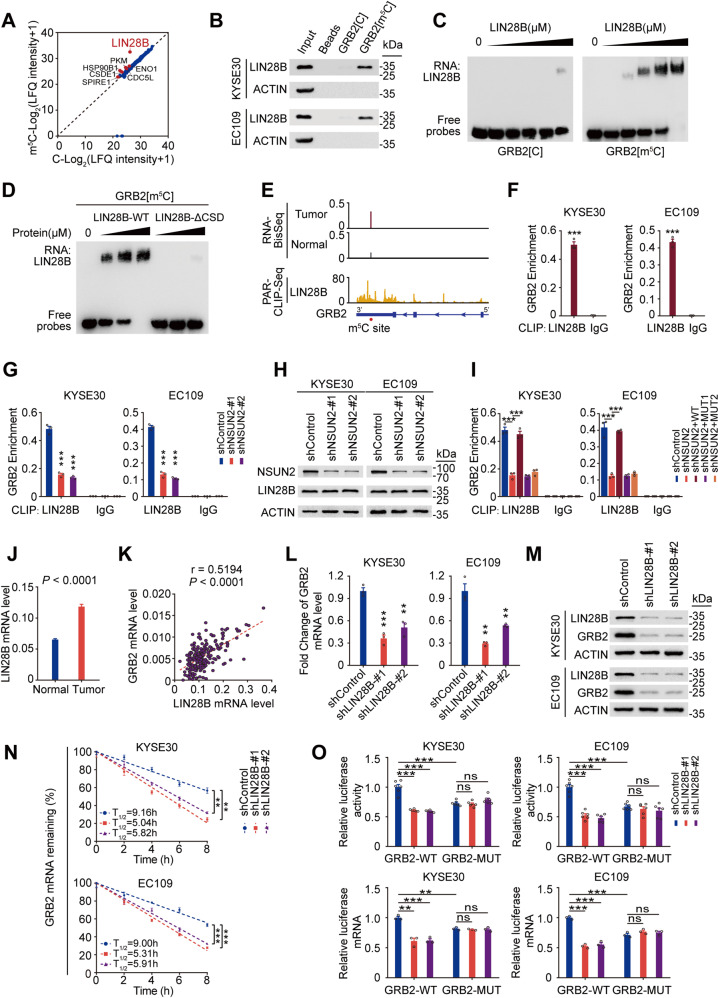
LIN28B stabilizes *GRB2* mRNA by recognizing its m^5^C site. **A** Scatter plot of proteins binding to 50 bp *GRB2* probe with or without m^5^C modification in KYSE30 cells. The filled red circles indicate proteins preferentially binding to *GRB2*[m^5^C] probes. **B** Western blotting analysis of potential *GRB2*[m^5^C] binding proteins obtained from RNA pull-down assays with 50 bp *GRB2* probe with or without m^5^C modification shows specific association of LIN28B with *GRB2*[m^5^C] probes. **C** RNA Electrophoretic mobility shift (REMSA) assays of purified FLAG-tagged LIN28B with unmethylated or methylated *GRB2* probes. The probes were maintained constantly while a gradient of 0–8 μM purified LIN28B was added to the reactions. **D** REMSA assays of *GRB2*[m^5^C] probes with purified FLAG-tagged LIN28B (wild-type or CSD domain truncation mutants). **E** Integrative-genomics-viewer (IGV) profiles showing the m^5^C levels of *GRB2* in tumors and adjacent normal samples as well as the LIN28B-binding groups in PAR-CLIP-Seq data. The filled red circle represents the m^5^C site (chr17:75318971) in *GRB2*. **F**–**G** PAR-CLIP-qPCR assays showing direct in-vivo binding of LIN28B to *GRB2* in ESCC cells (**F**), and significant reduction of LIN28B binding to *GRB2* when *NSUN2* was silenced (**G**). **H** *NSUN2* depletion had no influence on LIN28B protein levels. **I** Wild-type but not mutant *NSUN2* reversed the reduction of LIN28B binding to *GRB2* caused by *NSUN2* depletion. **J** *LIN28B* RNA levels were significantly higher in ESCC tumors than in paired normal tissues (*n* = 215, SYSUCC cohort). **K** Spearman’s correlation analysis between *LIN28B* and *GRB2* RNA levels in ESCC tumors (*n* = 215). **L**–**M** *LIN28B* knockdown substantially decreased the RNA (**L**) and protein (**M**) levels of *GRB2*. **N** Effects of *LIN28B* knockdown on mRNA half-life of *GRB2* by RNA stability assays. **O** Luciferase reporter assays of luciferase reporter gene with wild-type *GRB2*-m^5^C site (GRB2-WT) or mutant m^5^C site (GRB2-MUT) in the control or stable *LIN28B* knockdown ESCC cells. Data in (**F**), (**G**), (**I**), (**J**), (**L**), (**N**) and (**O**) are displayed as mean ± SEM. All data are from at least three independent experiments. *P* values are calculated by two-sided Student’s *t* test (*P < 0.05, **P < 0.01 and ***P < 0.001. ns not significant) in (**F**), (**G**), (**I**), (**L**), (**N**) and (**O**), and by two-sided paired Wilcoxon signed-rank test in (**J**). IgG served as an isotype control in (**F**), (**G**) and (**I**). ACTIN served as a control in (**B**), (**H**) and (**M**).

It is known that RNA methylation regulates its target RNA by reader proteins [28]. Since m^5^C modification could stabilize *GRB2* mRNA, we first examined the effect of the known reader YBX1 on *GRB2* and found that silencing *YBX1* had no influence on *GRB2* levels (Supplementary Fig. 6A). To identify the m^5^C mediators involved in *GRB2* regulation, we performed mass spectrometry analysis of proteins obtained by RNA pulldown using biotin-labelled 50-bp *GRB2* or m^5^C-*GRB2* RNA probes (Supplementary Data 2). We identified seven proteins preferentially binding to m^5^C-*GRB2* probes (Fig. 6A; Supplementary Fig. 6B), and among which, only LIN28B was verified by western blotting (Fig. 6B; Supplementary Fig. 6C) and REMSA assays (Fig. 6C). Since LIN28B has a conserved cold shock domain (CSD) similar to YBX1 [29], an m^5^C reader targeting m^5^C-modified RNAs through its CSD domain [12, 13], we hypothesis that LIN28B binds m^5^C-modified *GRB2* through CSD domain. REMSA experiment confirmed this notion that truncation of LIN28B CSD domain (LIN28B-ΔCSD) led to reduction in binding affinity of LIN28B toward the m^5^C-containing *GRB2* oligo compared with full-length LIN28B (LIN28B-WT) (Fig. 6D; Supplementary Fig. 6D). To further screen key residues for LIN28B to bind to m^5^C site of *GRB2*, we performed structural modeling for LIN28B in complex with the m^5^C containing *GRB2* RNA hexamer oligo using YBX1-m^5^C RNA complex as a reference model [12]. According the modeled LIN28B-m^5^C RNA complex structure, W36, M41, F43, D61, H65, and K92 might be the key interacting residues (<3 angstrom), and N38 and D61 had polar contacts with m^5^C RNA fragment. Furthermore, the residue interaction network generated by RING software [30] showed that there was Van der Waals force between the W36 residue and m^5^C, which indicated that W36 was critical for the LIN28B-m^5^C RNA interaction (Supplementary Fig. 6E−G). REMSA assays further confirmed W36 as the key residue for LIN28B to distinguish and bind to m^5^C-modified *GRB2* RNA (Supplementary Fig. 6D; Supplementary Fig. 6H). LIN28B-PAR-CLIP further showed direct binding of LIN28B to *GRB2* m^5^C site (Fig. 6E, F). This interaction was reduced in *NSUN2*-silenced cells (Fig. 6G) where LIN28B protein level was not altered (Fig. 6H) and overexpressing wild-type but not mutant *NSUN2* could reverse this reduced interaction (Fig. 6I). In addition, we found that *LIN28B* RNA levels were upregulated in ESCC tumors and were positively associated with *GRB2* RNA levels (*n* = 215; Fig. 6J, K; Supplementary Table 1). Since LIN28B could bind and stabilize its target RNAs [31], we assumed that NSUN2-enhanced *GRB2* stability depends on LIN28B. As expected, *LIN28B* silencing markedly decreased mRNA (Fig. 6L) and protein (Fig. 6M) levels of *GRB2*, and also reduced *GRB2* mRNA stability (Fig. 6N). In addition, luciferase expression of GRB2-WT was strongly decreased in *LIN28B*-knockdown cells, whereas GRB2-MUT had no such effects (Fig. 6O). These findings suggest that LIN28B is a novel m^5^C mediator recognizing the m^5^C-modified *GRB2* and stabilizing *GRB2* mRNA.

We then evaluate the LIN28B binding to other m^5^C-modified RNAs. A substantial decrease of LIN28B RNA-binding affinity was observed in ESCC cells when *NSUN2* was silenced (Supplementary Fig. 6I). By further analyzing RNA-BisSeq and LIN28B-PAR-CLIP-Seq data, we observed a substantial overlap of m^5^C-modified RNAs and LIN28B-binding target RNAs (Supplementary Fig. 6J). Moreover, ~29% of the m^5^C sites were localized within the LIN28B peaks (Supplementary Fig. 6K). In addition, a significant enrichment of m^5^C modifications in LIN28B-bound RNAs was observed (Supplementary Fig. 6L). These findings indicate that LIN28B may also recognize m^5^C-modified RNAs other than *GRB2*.

### GRB2 serves as an oncogene in ESCC, and the NSUN2-GRB2 axis is clinically relevant to ESCC

**Fig. 7:**
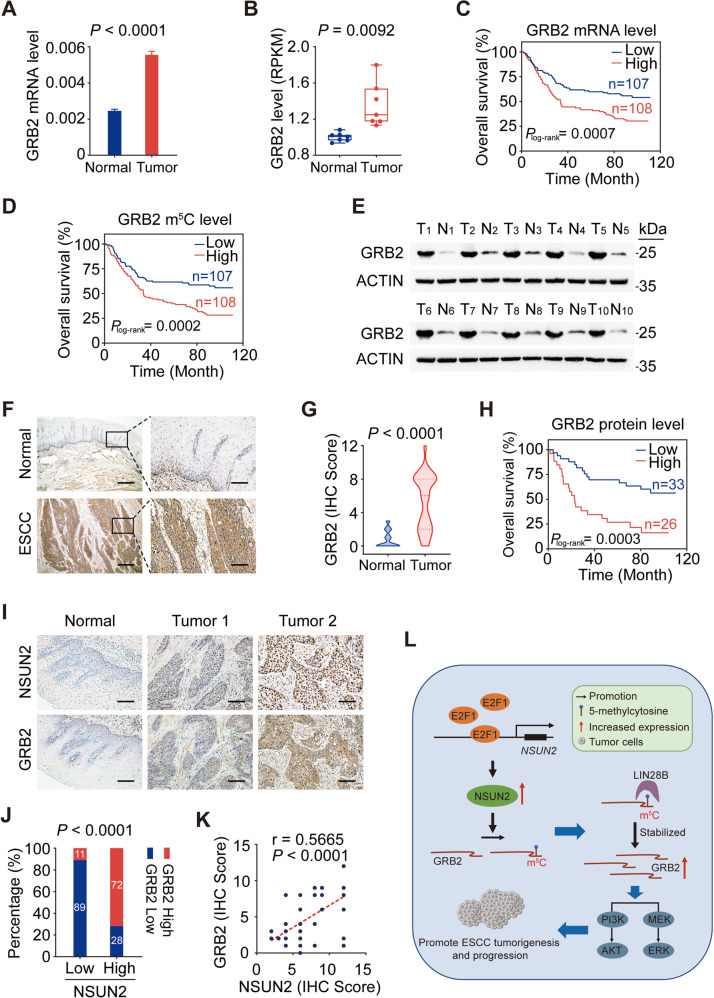
Clinical significance of NSUN2-GRB2 axes in ESCC. **A**, **B** Aberrant overexpression of *GRB2* RNA in ESCC tumors than in paired normal tissues by qRT-PCR (*n* = 215; **A**) and by RNA-Seq (*n* = 7; **B**). **C, D** Kaplan–Meier estimates of survival time of patients in SYSUCC cohort (*n* = 215) by different *GRB2* RNA levels (**C**) or m^5^C levels (**D**) in tumors. Median survival time for patients with high *GRB2* RNA or m^5^C levels (≥median) was 33.6 months or 33.6 months compared with 85.9 or 88.4 months in patients with low *GRB2* RNA (**C**) or m^5^C levels (**D**) (<median), with the adjusted HRs of 1.78 (95% CI, 1.22–2.61) and 1.70 (95% CI, 1.16–2.49), respectively. **E** Western blotting assays showing higher protein levels of *GRB2* in ESCC tumors than in paired normal tissues (*n* = 10). **F** Representative immunohistochemical staining (IHC) images of GRB2 protein in ESCC tumors and in paired normal tissues. Scale bar, 500 μm (left panel) and 100 μm (right panel). **G** GRB2 protein levels were higher in ESCC tumors than in paired normal tissues (*n* = 59) as indicated by the IHC score. **H** Kaplan–Meier estimates of survival time of patients by different GRB2 protein levels in tumors. The median survival time for patients with high GRB2 (IHC score > 6) was 22.8 months compared with 85.4 months in patients with low GRB2 (IHC score ≤ 6), with the adjusted HRs of 3.64 (95% CI, 1.57–8.41). **I** Representative images showing positive correlations between protein levels of *NSUN2* and *GRB2* in ESCC specimens. Scale bar, 100 μm. **J** Statistical analysis of IHC staining showing the percentage of ESCC specimens displaying higher or lower NSUN2 levels and the corresponding GRB2 expression. **K** Spearman’s correlation analysis between protein levels of *NSUN2* and *GRB2* in ESCC tumors (*n* = 59). **L** A proposed model for the regulatory mechanism of E2F1-NSUN2-m^5^C/LIN28B-GRB2-PI3K/AKT and ERK/MAPK signaling axes in the tumorigenesis and progression of ESCC. Data represent as mean ± SEM in (**A**), boxplots in (**B**) and violin plots in (**G**). The centerlines of the boxplots and violin plots represent median, while the upper and lower hinges indicate 25th and 75th percentiles, respectively. *P* values were calculated using the two-sided paired Wilcoxon signed-rank test in (**A**) and (**G**), two-sided paired *t*-test in (**B**), two-sided Chi-square test in (**J**) and two-sided log-rank test in (**C**), (**D**) and (**H**). ACTIN served as a control in (**E**).

We further investigate the role of *GRB2* in ESCC. Functionally, *GRB2* knockdown dramatically suppressed cell proliferation, migration, and invasion (Supplementary Fig. 7A, B). Clinically, we found higher m^5^C and RNA levels of *GRB2* in tumors than in adjacent normal tissues from our 215 paired ESCC cohort (Fig. 4F; Fig. 7A; Supplementary Table 1−2) and from the seven paired sequencing ESCC samples (Fig. 4G; Fig. 7B; Supplementary Table 7). Furthermore, patients with high *GRB2* mRNA or m^5^C levels had shorter OS than those with low levels (Fig. 7C, D; Supplementary Table 3). Consistently, GRB2 protein levels were significantly higher in ESCC tumors than in paired normal tissues detecting by western blotting (*n* = 10; Fig. 7E) and IHC assays (*n* = 59; Fig. 7F, G; Supplementary Table 4−5). Patients with higher GRB2 protein level also had worse prognosis (Fig. 7H; Supplementary Table 6). These findings indicate that *GRB2* was an independent prognostic factor in ESCC.

Finally, we explored the clinical importance of the NSUN2-GRB2 axis in ESCC. As expected, overexpressing *GRB2* rescued the inhibition of malignant phenotypes in *NSUN2*-depleted cells (Supplementary Fig. 7C, D). Clinically, approximately 89% of the specimens with lower NSUN2 protein level presented weaker IHC staining of GRB2, while nearly 72% of the specimens with higher NSUN2 showed stronger IHC staining of GRB2 (Fig. 7I, J). Spearman’s correlation analysis also revealed a positive correlation between protein level of *NSUN2* and *GRB2* (Fig. 7K). These results demonstrate the clinical correlation between *NSUN2* and *GRB2*, and reveal an oncogenic role of the NSUN2-GRB2 axis in ESCC.

## Discussion

RNA m^5^C modification is a common posttranscriptional RNA modification participating in many cellular and physiological processes [32]. Abnormal m^5^C modification is associated with various diseases, including cancer [12, 33], inflammation [14], intellectual disabilities [22], neurodevelopmental disorders [34], infertility [35], and mitochondrial dysfunction [36]. However, the precise correlations between RNA m^5^C modification and tumor development remains largely unclear. In this study, we described a transcriptome-wide m^5^C profile in ESCC for the first time, which showed aberrantly increased levels of RNA m^5^C modification in ESCC tumors due to the overexpression of *NSUN2*. *NSUN2* plays a critical role in ESCC by positively regulating *GRB2* through the m^5^C-LIN28B-based posttranscriptional regulation, while its own transcription is positively regulated by E2F1. Increased expression of *GRB2* promoted the development and progression of ESCC by triggering the abnormal activation of PI3K/AKT and ERK/MAPK signaling (Fig. 7L).

RNA m^5^C modification is catalyzed by several methyltransferases [11], among which, NSUN2 has attracted increasing attentions because of its oncogenic role in various types of cancers [12, 23, 37–39]. Our results in this study are consistent with previous findings and extend the oncogenic role of *NSUN2* to ESCC. We found aberrant overexpression of *NSUN2* in ESCCs, which was highly associated with poor prognosis and advanced tumor stages, suggesting a prognostic value for *NSUN2*. In-vitro experiments showed that *NSUN2* promoted malignant phenotypes of ESCC cells dependent on its methyltransferase activity, suggesting an m^5^C-dependent oncogenic role of *NSUN2*. In according with in-vitro data, *NSUN2* silencing markedly suppressed ESCC tumor initiation and progression in the 4-NQO-induced ESCC model in transgenic mice, indicating that *NSUN2* overexpression may be an early molecular event of ESCC. Our findings demonstrate that *NSUN2* is essential for the malignancy of ESCC in an m^5^C-dependent manner. As a previous study [21] has indicated that NSUN2 is not expressed in quiescent stem and undifferentiated progenitor cells but highest expressed in committed progenitors in mouse skin squamous cell carcinoma model, it would be interesting to investigate differences in tumor cell population ratios between different tumor models.

Another interesting finding is the identification of E2F1 as an *NSUN2* transcriptional activator. In this study, we found that overexpression of *NSUN2* in ESCC was not caused by genomic changes as indicated by no significant alterations of mutations, CNV and DNA methylation status at *NSUN2*. Integrated analysis suggests that E2F1 was a potential regulator of *NSUN2*. E2F1 is a well-known TF that has been shown to be aberrant expressed in ESCC tumors [40]. Consistently, in our study, we demonstrated that E2F1 was upregulated in ESCC tumors and was positively correlated to *NSUN2* expression, supporting a positive regulation of *NSUN2* expression by E2F1. Moreover, we demonstrated that E2F1 could bind to the promoter region of *NSUN2*, stimulating *NSUN2* transcription. These findings reveal for the first time that overexpression of *NSUN2* in ESCC may be partially mediated by trans-element(s).

To further address the oncogenic function of NSUN2-mediated m^5^C methylation in ESCC, we provided an RNA m^5^C landscape in ESCC and demonstrated an oncogenic role of RNA m^5^C-hypermethylation in ESCC. Notably, genes with hypermethylated-m^5^Cs in ESCC tumors were significantly enriched in PI3K/AKT and ERK/MAPK pathways. It is known that both PI3K/AKT and ERK/MAPK pathways play vital roles in various cellular processes [25, 41]. Dysfunction of both pathways is involved in many diseases such as malignant tumors [25, 41]. Several studies have reported that ESCC patients show abnormal activation of PI3K/AKT and ERK/MAPK signaling, which may provide useful therapeutic targets of ESCC [42, 43]. However, how these two pathways are activated in ESCC and by what remains largely unclear. In the present study, we demonstrated that aberrant expression of *NSUN2* could trigger abnormal activation of PI3K/AKT and ERK/MAPK signaling via stabilizing *GRB2* mRNA. It has been reported that GRB2, as a growth factor receptor-bound protein, is involved in various biological processes, such as cell growth, proliferation, and metabolism [26]. GRB2 plays a central role between cell-surface activation receptors (RTK, integrin, etc.) and other downstream signaling pathways, especially PI3K/AKT and ERK/MAPK signalling [26, 27, 44]. Abnormal expression of *GRB2* promotes tumor malignancies by activating both PI3K/AKT and ERK/MAPK pathways [26, 27, 44]. Here, we have linked aberrant m^5^C modifications in *GRB2* to the development and progression of ESCC via PI3K/AKT and ERK/MAPK pathways. *NSUN2* silencing attenuated PI3K/AKT and ERK/MAPK signaling via decreasing both m^5^C and mRNA levels of *GRB2*, while *GRB2* overexpression reversed the inhibition of malignant cellular phenotypes in *NSUN2*-depleted cells, suggesting that *GRB2* is a key mediator of malignancy induced by abnormal NSUN2-meidated m^5^C modification in ESCC. Our results suggest the central role of NSUN2-m^5^C-GRB2-PI3K/AKT and ERK/MAPK axes to the pro-tumorigenic effect of *NSUN2* in ESCC and provide a new acting model for NSUN2-mediated regulation of ESCC progression. Moreover, a number of reports have indicated that *GRB2* overexpression is associated with poor prognosis in cancers [26] including ESCC [45]. Our results are in accordance with these studies showing that both mRNA and protein level of *GRB2* were upregulated in ESCC tumors and high level of *GRB2* predicted poor prognosis. Furthermore, *GRB2* overexpression was previously indicated to be related to lymph node metastasis in ESCC [45], indicating *GRB2* would play critical roles in metastasis in ESCC. This observation supports the results of our study showing that *GRB2* promotes invasion and metastasis in ESCC cells. Until now, mechanisms controlling the expression of *GRB2* remain unclear. Regulation of *GRB2* by miRNAs binding to its 3′UTR such as miR-433-3p was previously shown in ESCC [46], suggesting that targeting *GRB2*-asscocited miRNAs may have therapeutic benefits in ESCC. In addition, a potential antitumor drug F806 was previously reported to downregulate *GRB2* level to inhibit cellular proliferation signaling network in ESCC [47]. All these findings suggest that reducing the *GRB2* level could arrest the malignant growth of cancers, which may provide a potential treatment strategy for ESCC. In our current research, we have shown that the m^5^C site in the 3′UTR of *GRB2* RNA plays critical roles in *GRB2* upregulation through enhancing *GRB2* mRNA stability. Our findings have extended the regulatory mechanism of *GRB2* expression from an epigenetic perspective and revealed a new mechanism for regulating *GRB2* expression through m^5^C modification, indicating that downregulation of m^5^C modification in *GRB2* may also have therapeutic benefits in ESCC via decreasing *GRB2* expression. As GRB2 has been suggested to be a therapeutic target for cancers by targeting its SH2/SH3 domains [48, 49], it may be interesting to further study the synergistic anticancer effect of GRB2 and NSUN2-mediated m^5^C modification in ESCC. Since other oncogenic transcripts, such as *PIK3R3*, which has been previously shown to trigger the activation of the PI3K/AKT pathway in ESCC cells [50], also exhibit abnormal m^5^C modification, it would be interesting to further explore whether m^5^C modification has the same regulatory function as *GRB2* on various transcripts or cancer-related signaling.

Another interesting finding is a new RNA m^5^C mediator. The biological importance of RNA methylation relies on reader proteins [28]. Previous studies have reported ALYREF and YBX1 as m^5^C readers [9, 12, 13]. In our study, we showed an m^5^C-mediated RNA stabilization of *GRB2*, which was YBX1-independent. Then, by mass spectrometry analysis, we have showed that LIN28B preferentially interacts with m^5^C-modified *GRB2*. This notion is further confirmed by several experiments showing that the binding of LIN28B to *GRB2* is m^5^C-dependent. LIN28B is a known oncoprotein aberrantly expressed in a subset of human cancers [51], including ESCC [52], which is consistent with our results. It contains a conserved CSD domain [29], which is necessary for YBX1 binding to m^5^C-modified RNAs [12]. In our study, we have identified a critical role of CSD domain for LIN28B binding to m^5^C-carrying *GRB2*. By performing structure modeling analysis and in-vitro RNA-protein interaction assays, we have identified the LIN28B as an m^5^C mediator preferentially recognizing m^5^C-carrying *GRB2* RNA through the W36 residue in its CSD domain. The mode of LIN28B binding to m^5^C-modified *GRB2* RNA is extremely similar to YBX1, which binds to m^5^C-modified RNAs through the indole ring of W65 in its CSD [12]. However, this binding mechanism is different from that of ALYREF, which recognizes and interacts with m^5^C-modified RNAs mainly through a conserved positively charged residue, K171 [9]. It has been shown that LIN28B regulates RNA stability mainly through inhibiting let-7 microRNAs biogenesis [53] or through directly binding to its target RNAs [31, 54]. As our observations suggest, LIN28B could indeed stabilize GRB2 transcripts. However, this stabilization is m^5^C-dependent, which might be a novel mechanism of LIN28B-dependent regulation to its target RNAs. Our results strongly indicate that LIN28B is likely an m^5^C mediator stabilizing m^5^C-modified *GRB2*. This function is similar to YBX1 that plays an essential role in maintaining m^5^C-carrying RNA stability [12] but not ALYREF that exerts an RNA-export-promoting function through recognizing m^5^C-modified RNAs [9]. Since the m^5^C modification was located in 3′UTR of *GRB2* RNA, which was previously shown to be bound by miR-433-3p in ESCC [46], it would be worth exploring the association between m^5^C modification and miRNA binding in *GRB2* 3′UTR. Previous study has suggested that presence of RNA m^6^A methylation on some transcripts could affect the RBP-RNA interactions and the miRNA targeting in 3′UTR region of RNAs [55], whether m^5^C modification exerts similar regulatory function remains to be further clarified. Although we have also shown the overlap of LIN28B-binding targets and m^5^C-modified RNAs, whether LIN28B is a common reader and the exact molecular mechanism for the association of LIN28B and other m^5^C-modified RNAs are warranted to further investigation.

In conclusion, our current work elucidates a vicious role of the NSUN2-m^5^C-GRB2-PI3K/AKT and ERK/MAPK signaling axes in the initiation and the progression of ESCC. These findings illustrate an m^5^C-mediated epigenetic regulation mechanism of ESCC and highlight the opportunity for epitranscriptomic-targeted therapy for ESCC.

## Materials and methods

### Patient sample collection

Written informed consent was obtained from each patient, and this study was approved by the Institutional Review Board of the SYSUCC.

### Cell lines and cell culture

Human ESCC cell lines KYSE30 and EC109 were kind gifts from Dr. Xinyuan Guan at SYSUCC.

### Cell proliferation, migration and invasion assays

Details of in-vitro functional experimental procedures could see in Supplementary Materials and methods.

### RNA extraction and quantitative real-time PCR (qRT-PCR)

The primer sequences are shown in Supplementary Table 8.

### Western blotting analysis

Antibodies against the interest proteins are shown in Supplementary Table 9.

### Chromatin immunoprecipitation (ChIP) assays

Specific primers used are listed in Supplementary Table 8. qPCR products were used for agarose gel electrophoresis.

### 4-NQO-induced ESCC model in *Nsun2* knockout transgenic mice

*Nsun2* knockout (*Nsun2*+/−) or *Nsun2* wild-type (*Nsun2*+/+) C57BL/6J mice were donated from Nanjing Medical University. Animal experiments were carried out with protocols and guidelines approved by the Institutional Animal Care and Use Committee of SYSUCC.

### Construction of RNA-BisSeq and RNA-Seq libraries

The procedure was performed according to a previous study with some modifications [9]. Analysis of the *Dhfr* spike-in showed C to T conversion rates >99%.

### Differential m^5^C methylation analysis

M^5^C sites with *P* ≤ 0.05 and a mean m^5^C level difference ≥0.05 (|mean m^5^C level _tumor_ − mean m^5^C level _normal_|) were considered to contain statistically significantly different m^5^C methylation.

### Pathway analysis via Ingenuity Pathway Analysis (IPA)

Genes with m^5^C-hypermethylated transcripts were uploaded into IPA software for core analysis to identify canonical pathways (FDR ≤ 0.1) [56].

### M^5^C RNA immunoprecipitation followed by qRT-PCR (m^5^C-RIP-qPCR)

The m^5^C-RIP-qPCR procedure was performed according to a previous study with some modifications [13]. Gene-specific primers are shown in Supplementary Table 8. Relative m^5^C levels of the indicated transcripts were evaluated with input normalization.

### Photoactivatable-ribonucleoside-enhanced crosslinking and immunoprecipitation (PAR-CLIP)

Cells were cultured in medium supplemented with 4-thiouridine for 14 h, followed by UV light crosslinking. The relative enrichment of the interest transcripts was calculated with input normalization.

### RNA interference

Small interfering RNA (siRNA) targeting *TFAP2C*, *SP1*, *NRF1*, *E2F1* or *YBX1* genes are listed in Supplementary Table 10.

### Plasmids, lentivirus production and transduction

Short hairpin RNA specifically targeting *NSUN2*, *LIN28B* or *GRB2* are listed in Supplementary Table 10.

### RNA stability assay

Cells were treated with actinomycin D and the mRNA half-life time was calculated as previously described [57].

### Luciferase reporter gene assays

The luciferase activity or RNA level was examined using the Dual-Luciferase Reporter Assay System (Promega, Madison, WI, USA) or qRT-PCR, respectively.

### RNA pulldown and mass spectrometry analysis

Biotin-labeled RNA fragment containing 50 bp *GRB2* RNA sequences with (*GRB2*[m^5^C]) or without (*GRB2*[C]) m^5^C modification at m^5^C site (chr17:75318971) were listed in Supplementary Table 10.

### RNA electrophoretic mobility shift assays (REMSA)

Assays were performed using the LightShift Chemiluminescent RNA EMSA Kit (Thermo Fisher Scientific, Waltham, MA, USA).

### Public data processing

Details are described in Supplementary Materials and methods.

### Statistical analysis

All the statistical analyses were performed using SPSS version 20.0 software (SPSS Inc., Chicago, IL, USA) or GraphPad Prism 8.0 software (GraphPad Software, La Jolla, CA, USA). *P* < 0.05 was considered statistically significant.

### Supplementary materials and methods

For the details of other experimental methods, see the Supplementary Materials and methods.

## Supplementary information

Supplementary Information files

Supplementary Data 1

Supplementary Data 2
