# Supplementary material for: NSUN2-mediated RNA 5-methylcytosine promotes esophageal squamous cell carcinoma progression via LIN28B-dependent GRB2 mRNA stabilization
Source: Oncogene. 2021 Aug 3;40(39):5814–28. doi: 10.1038/s41388-021-01978-0 (PMC8484015; doi:10.1038/s41388-021-01978-0)
Supplement: Supplementary file 1 — Supplementary Information files [file 41388_2021_1978_MOESM1_ESM.pdf]

Supplementary Information files

NSUN2-mediated RNA 5-methylcytosine promotes esophageal squamous cell carcinoma progression via LIN28B-dependent GRB2 mRNA stabilization

Su *et al*.

Supplementary Materials and methods

Supplementary Figures

Supplementary Fig. 1: Expression levels of *NSUN2* and *NSUN6* in patients with ESCC. Related to Fig. 1.
Supplementary Fig. 2: Correlation between expressions of potential transcription factors and *NSUN2* at mRNA levels. Related to Fig. 2.
Supplementary Fig. 3: Effects of NSUN2 on ESCC cell migration and invasion. Related to Fig. 3.
Supplementary Fig. 4: Validation of *Nsun2* knockout mice. Related to Fig. 3.
Supplementary Fig. 5: Distribution profiles of RNA m^5C modification in human ESCC. Related to Fig. 4.
Supplementary Fig. 6: Validation of the binding abilities of potential *GRB2*[m^5C] binding proteins to methylated or unmethylated *GRB2* probes. Related to Fig. 6.
Supplementary Fig. 7: Effects of NSUN2-GRB2 axis on malignant cell phenotypes of ESCC cells. Related to Fig. 7.

Supplementary Tables

Supplementary Table 1: Baseline demographic and clinical characteristics of individuals with ESCC used for qRT-PCR and m^5C-RIP-qPCR in this study.
Supplementary Table 2: Associations between *NSUN2*, *GRB2* RNA level or *GRB2* m^5C level and clinical characteristics of individuals with ESCC used for qRT-PCR and m^5C-RIP-qPCR in this study.
Supplementary Table 3: Univariate and multivariate Cox regression analysis for overall survival in ESCC patients.
Supplementary Table 4: Baseline demographic and clinical characteristics of individuals with ESCC used for IHC in this study.
Supplementary Table 5: Associations between *NSUN2*, *GRB2* protein level and clinical characteristics of individuals with ESCC used for IHC in this study.
Supplementary Table 6: Univariate and multivariate Cox regression analysis for overall survival in ESCC patients.
Supplementary Table 7: Characteristics of ESCC individuals for RNA-BisSeq and RNA-Seq in this study.
Supplementary Table 8: Primers used in this study.
Supplementary Table 9: Antibodies utilized in this study.
Supplementary Table 10: Targeted sequences of shRNAs or siRNAs and probe sequences of *GRB2* used in this study.

Description of Additional Supplementary Files

File Name: **Supplementary Data 1**
Description: m^5C sites with differential methylation level identified in RNA-BisSeq of 7 paired ESCC in this study.
File Name: **Supplementary Data 2**
Description: Proteins identified in RNA pulldown and mass spectrometry analysis with *GRB2*[m^5C] or *GRB2*[C].

Supplementary Materials and methods

Patient sample collection

This study consisted of two sets of ESCC patients: one set of 7 paired samples for RNA-BisSeq and RNA-Seq analyses collected from 2015−2017 (**Supplementary Table 7**) and another set of 215 paired samples for verification of the sequencing results collected from 2012−2014 (**Supplementary Table 1**). All tissue specimens used in this study were obtained during esophageal cancer resection surgery at Sun Yat-sen University Cancer Center (SYSUCC) and were immediately stored in liquid nitrogen until RNA or protein extraction. ESCC diagnosis was histopathologically confirmed by at least 3 pathologists and clinical characteristics of each patient were obtained from medical records. Follow-up data were obtained by accessing medical records, telephone calls or outpatient visits. Patient's overall survival (OS) time was measured from the date of tumor diagnosis to the date of last follow-up or death. Tumor stages were defined according to the 7th edition of the AJCC Cancer Staging System [1]. Among the 215 paired ESCC samples, randomly selected 10 or 59 paired samples (**Supplementary Table 4**) were respectively used for western blotting or immunohistochemical staining (IHC). Written informed consent was obtained from each patient, and this study was approved by the Institutional Review Board of the Sun Yat-sen University Cancer Center.

Cell lines and cell culture

Human ESCC cell lines KYSE30 and EC109 were kind gifts from Dr. Xinyuan Guan at SYSUCC. Human embryonic kidney cell line 293T (HEK293T) was purchased from the Cell Bank of Type Culture Collection of the Chinese Academy of Sciences Shanghai Institute of Biochemistry and Cell Biology. Cells were cultured in DMEM (HEK293T) or RPMI 1640 (all other cell lines)

medium supplemented with 10% fetal bovine serum (FBS, Invitrogen, Carlsbad, CA, USA) in an atmosphere of 5% CO_2 and 99% relative humidity at 37°C. All cell lines were authenticated by STR profiling and tested for free from mycoplasma infection.

Cell proliferation, migration and invasion assays

For cell proliferation assays, KYSE30 and EC109 cells were seeded in 96-well plates (3,000 cells per well). Cell viability was measured at indicated time points using Cell Counting Kit-8 (CCK-8, Dojindo Laboratories, Kumamoto, Japan). Each experiment was repeated three times, with six replicates each time. For migration assays, 8-µm pore inserts were placed in a 24-well culture plate. 1×10^5 cells in 200 µl of serum-free medium were added into the upper filters. 500 µl medium containing 20% FBS was added to the lower chamber. After incubation for 15 h in 5% CO_2 at 37°C, cells migrated through the filters were fixed with methanol, stained with 0.5% crystal violet and photographed. Cells in three random fields were counted. Invasion assays were conducted following a similar protocol with coating filters with 30 µg of matrigel (BD Biosciences, San Diego, CA, USA). Each experiment was performed three times.

RNA extraction and quantitative real-time PCR (qRT-PCR)

Total RNA was isolated from cell lines or tissues using TRIzol reagent (Invitrogen, Carlsbad, CA, USA). Reverse transcription reactions with 2 µg total RNA were performed using the RevertAid First-Strand cDNA Synthesis Kit (Thermo Fisher Scientific, Waltham, MA, USA). Synthesized cDNA was used for qRT-PCR analysis on a Roche Light Cycler 480 II using the SYBR-green method [2]. *β-ACTIN* was employed as an internal control. Relative RNA levels of indicated genes were calculated using the 2-ΔΔCT method and normalized to *β-ACTIN*. Three replicates

were performed in each experiment. The primer sequences are shown in **Supplementary Table 8**.

Cell lysis and protein immunoprecipitation

Cells were lysed with 1 × RIPA buffer supplemented with a Protease/Phosphatase Inhibitor Cocktail (Pierce, USA). Lysates were centrifuged. Supernatants were treated with RNase inhibitor (New England Biolab, Ipswich, MA, USA) and collected for immunoblotting or immunoprecipitation with the indicated antibodies.

Western blotting analysis

Protein extracts were prepared using 1 × RIPA buffer supplemented with a Protease/Phosphatase Inhibitor Cocktail (Pierce, USA). Total protein (20 μg) was subjected to SDS-PAGE and transferred to PVDF membranes (Millipore, Billerica, MA, USA). Membranes were incubated overnight at 4°C with specific primary antibody (**Supplementary Table 9**) and visualized with a Phototope Horseradish Peroxidase Western Blot Detection kit (Thermo Fisher Scientific, Waltham, MA, USA).

Chromatin immunoprecipitation (ChIP) assays

ChIP assays were performed using the EZ-Magna ChIPTM A/G Kit (17-10086, Millipore). Briefly, cells were treated with 1% formaldehyde for cross linking, lysed and sonicated on ice. Lysates were then immunoprecipitated with antibody against E2F1 (66515-1-Ig, Proteintech) or IgG. Pre-immunoprecipitated lysates of each sample were saved as input. The bound DNA was eluted, purified and subject to qPCR with specific primers (**Supplementary Table 8**). qPCR products were used for agarose gel electrophoresis.

Chemicals

Chemical carcinogen 4-NQO (N8141) and 2% propylene glycol (V900115) were purchased from Sigma-Aldrich (St. Louis, MO, USA).

4-NQO-induced ESCC model in *Nsun2* knockout transgenic mice

Chemical carcinogen 4-NQO was used to establish mouse model of ESCC in *Nsun2* knockout (*Nsun2*+/-) or *Nsun2* wild-type (*Nsun2*+/+) C57BL/6J mice donated from Nanjing Medical University. To generate the *Nsun2*+/- mice, Cas9 mRNAs and two small guide RNAs targeting the fourth exon of *Nsun2* were injected into fertilized eggs, resulting in a 14-bp frameshift mutation on one allele of *Nsun2*. DNA extracted from mouse tails was used for Sanger sequencing to validate the *Nsun2* genotypes. Six-week-old *Nsun2*+/+ and *Nsun2*+/- mice were given 100 µg/ml 4-NQO in the drinking water for 16 weeks followed by normal water feeding [3]. 4-NQO was prepared in propylene glycol as a 5 mg/ml stock solution once a week and used at a 1:50 dilution in drinking water. The mice were grouped randomly. Some *Nsun2*+/+ mice were sacrificed 4 weeks (n = 3) and 8 weeks (n = 3) after 4-NQO withdrawal, and some (n = 10) were sacrificed along with age-matched *Nsun2*+/- mice (n = 10) 12 weeks after 4-NQO withdrawal. The other mice (n = 10 per group) were monitored to determine the survival rate. Immediately after the death of the mice, the esophagus was stripped, cut open longitudinally, spread flat and photographed. Tumors with diameters ≥ 0.5 mm were counted and tumor volume (length x width^2 x 0.5) was calculated. Esophagus was embedded in paraffin after 10% formalin buffer treatment or was used for protein extraction. Hematoxylin and eosin (H&E) staining was performed for histological examination. IHC or western blotting assay was conducted to assess levels of indicated protein. Pathological diagnosis was assessed by three pathologists blinded to the group allocation. To monitor survival, the differences in survival

time between *Nsun2*+/+ and *Nsun2*+/- mice were determined using Kaplan-Meier method. The animals that were euthanized for signs of illness or those that were found dead were included as events in the survival curve. Animal experiments were carried out with protocols and guidelines approved by the Institutional Animal Care and Use Committee of Sun Yat-sen University Cancer Center.

Sanger sequencing of PCR products

To validate the knockdown efficiency of *Nsun2* transgenic mice, DNA (1 μg) was extracted from mouse tails using the DNA Mini Kit (Qiagen, Hilden, Germany) and was amplified by PCR with the primers shown in **Supplementary Table 8**. PCR products were separated on an agarose gel and purified, followed by Sanger sequencing (Ruibiotech, Beijing, China).

Immunohistochemical staining (IHC) analysis

Paraffin-embedded tissue sections were selected for IHC analysis. Primary antibodies against NSUN2 (Proteintech, 20854-1-AP) and GRB2 (Abcam, ab32037) were used. Briefly, the sections were deparaffinized in xylene for 10 min twice and were rehydrated by sequentially incubation with 95%, 85% and 75% ethanol 5 min for each twice, followed by the heat-mediated antigen retrieval using microwave. After incubation in 3% H_2O_2 for 10 minutes, the slides were blocked with 5% goat blocking serum at room temperature for 30 min and incubated with the respective primary antibodies at 4°C overnight. Then, after washing, the slides were incubated with the biotinylated secondary antibody for 30 minutes at room temperature. Finally, the slides were incubated in ABC reagent for 30 min (the ABC Kit, Pierce, USA) and stained with DAB and counterstained with hematoxylin. The staining intensity score was defined as negative (0), weak (1), moderate (2) and strong (3) while the positive area score was estimated as 0 (≤

10%), 1 (11% to 25%), 2 (26% to 50%), 3 (51% to 75%) and 4 (> 75%). Both the staining intensity and positive area scores were independently assessed by three pathologists without prior knowledge of the patient data. IHC score (values from 0 to 12) was calculated by multiplying the staining intensity score and the positive area score. For esophageal tissues collected from the 4-NQO-treated mice, H&E was also performed.

Construction of RNA-BisSeq and RNA-Seq Libraries

Total RNA from ESCC specimens was used for mRNAs enrichment using a Dynabeads mRNA purification kit (Ambion, Austin, Texas, USA). For the RNA-BisSeq experiment, the enriched mRNAs alone with in-vitro-transcribed mouse *Dhfr* mRNA as methylation conversion control were fragmented and converted using bisulphite as previously described [4]. Briefly, 1 µg of the enriched mRNAs and 5 ng *Dhfr* mRNA were subject to fragmentation using the RNA fragmentation reagents (Ambion, Austin, Texas, USA). After ethanol precipitation, the fragmented RNAs were resuspended in 100 µl pre-prepared bisulfite solution (pH 5.1) and incubated at 75 °C for 4.5 h. The pre-prepared bisulfite solution is a mixture of hydroquinone (600 µM; Sigma-Aldrich, St. Louis, MO, USA) and sodium bisulfite (40%; Sigma-Aldrich, St. Louis, MO, USA) at a ratio of 1:100. Then the reaction product was desalted using the Micro Bio-spin 6 chromatography columns (Bio-Rad, Hercules, CA, USA) and desulfonated in Tris-HCl (1 M, pH 9.0) at 75°C for 60 min. The RNAs were precipitated with ethanol and reverse transcribed with ACT random hexamers and used for library construction. Each RNA-BisSeq sample was also subjected to an RNA sequencing assay. Sequencing was carried out on the Illumina HiSeq X-Ten sequencing system.

RNA-BisSeq bioinformatics analysis

We utilized fastp [5] to remove the adaptors and low-quality bases from the raw reads. The clean reads were mapped to the hg38 genome using meRanGh from meRanTK (v1.2.1b) [6]. Analysis of the *Dhfr* spike-in showed C to T conversion rates > 99%. The m^{5}C sites were called using meRanCall from meRanTK. Only the sites with a coverage depth ≥ 30, methylation level ≥ 0.1 and methylated cytosine depth ≥ 5 in at least 2 samples were considered reliable. The m^{5}C annotation was performed with intersectBed from BEDTools (v2.28.0) [7] with the annotation file (GENCODE v25) download from the GENCODE database [8].

Differential m^{5}C methylation analysis

To compare the methylation levels between ESCC tumor and adjacent normal samples, we used m^{5}C sites meeting the following criteria according to the previous study [9]: read coverage ≥ 10 in at least 8 samples, including ≥ 4 normal and ≥ 4 tumor samples. M^{5}C sites with $P \leq 0.05$ (two-sided unpaired Wilcoxon and Mann-Whitney tests) and a mean m^{5}C level difference ≥ 0.05 (|mean m^{5}C level$_{tumor}$ - mean m^{5}C level$_{normal}$|) were considered to contain statistically significantly different m^{5}C methylation. The hypermethylated m^{5}C sites in tumor samples were those with $P \leq 0.05$ and mean m^{5}C level$_{tumor}$ - mean m^{5}C level$_{normal}$ ≥ 0.05, while the hypomethylated m^{5}C sites in tumor samples were those with $P \leq 0.05$ and mean m^{5}C level$_{tumor}$ - mean m^{5}C level$_{normal}$ < -0.05.

RNA-Seq bioinformatics analysis

The raw reads of the RNA-Seq data were filtered with the same method as the RNA-BisSeq data and then mapped to the hg38 genome with hisat2(v2.1.0) [10]. HTSeq (v0.12.4) [11] was used to count the number of reads mapped to each gene (GENCODE v25). Differentially

expressed genes were calculated by the DESeq2 package [12] with |fold-change| ≥ 1.5 and false-discovery-rate (FDR) ≤ 0.05.

Pathway analysis via Ingenuity Pathway Analysis (IPA)

Genes with m^5C-hypermethylated transcripts were uploaded into IPA software for core analysis to identify canonical pathways (FDR ≤ 0.1) [13].

M^5C RNA immunoprecipitation followed by qRT-PCR (m^5C-RIP-qPCR)

The m^5C-RIP-qPCR procedure was performed according to a previous study with some modifications [14]. Briefly, total RNA from tissues or cells was purified into mRNAs using a Dynabeads mRNA purification kit (Ambion, Austin, Texas, USA). Then, the mRNAs (2 μg) were fragmented and incubated at 4°C overnight with the pre-mixture of anti-m^5C antibody and magnetic Dynabeads protein A/G (Millipore, Billerica, MA, USA) in RIP immunoprecipitation buffer (Millipore, Billerica, MA, USA). One-tenth of fragmented mRNAs were used as an input. After treating with proteinase K (10 mg/ml, New England Biolabs, Ipswich, MA, USA), the bound RNAs were extracted with phenol/chloroform/isoamyl alcohol and subjected to qRT-PCR using gene-specific primers shown in **Supplementary Table 8**. Relative m^5C levels of the indicated transcripts were evaluated with input normalization.

Photoactivatable-Ribonucleoside-Enhanced Crosslinking and Immunoprecipitation (PAR-CLIP)

PAR-CLIP was performed as previously described [15,16] with some modifications. Briefly, cells were cultured in medium supplemented with 4-thiouridine (Sigma-Aldrich, St. Louis, MO, USA) for 14 h, followed by crosslinking twice with UV light. Cells were lysed and sonicated. Then, the protein-RNA mixture was incubated and rotated overnight at 4°C with the premixture of

Dynabeads protein A/G (Millipore, Billerica, MA, USA) and anti-LIN28B antibody. The bound RNA was then treated with proteinase K (New England Biolabs, Ipswich, MA, USA) and extracted by phenol/chloroform/isoamyl alcohol, followed by 3' linker ligation. The NEBNext small RNA library prep kit (E7330S, New England Biolabs, Ipswich, MA, USA) was used for library construction of PAR-CLIP-Seq. Sequencing was performed on an Illumina HiSeq4000. For PAR-CLIP-qPCR, purified RNAs were reverse transcribed and subjected to qRT-PCR using primers shown in **Supplementary Table 8**. An equal amount of cell extract mixed with IgG was saved as an isotype control and one-tenth of cell extract was saved as an input control. The relative enrichment of the interest transcripts was calculated with input normalization. PAR-CLIP-biotin chemiluminescent nucleic acid detection were performed according to the previous study [17]. Briefly, the LIN28B bound RNAs were biotinylated using the Pierce™ RNA 3' End Desthiobiotinylation Kit (Thermo Fisher Scientific, Waltham, MA, USA). One tenth of the samples were subjected to western blotting assays to detect protein immunoprecipitation efficiency. Other samples were separated in native polyacrylamide gels and then transferred to a nylon transfer membrane, followed by visualized with the chemiluminescent nucleic acid detection Module Kit (Thermo Fisher Scientific, Waltham, MA, USA) or incubated with anti-m^5C antibody (Abcam, ab10805) to detect the m^5C level.

PAR-CLIP-Seq data analysis

Bowtie (v1.2.2) [18] was used to map sequencing reads against the hg38 genome with the parameters "-v 3 -m 5 --best –strata". PARalyzer (v1.5) [19] was used to define LIN28B binding groups with default parameters. The PAR-CLIP-Seq signal tracks were visualized using Integrative Genomics Viewer (IGV) [20].

RNA interference

Small interfering RNA (siRNA) targeting *TFAP2C*, *SP1*, *NRF1*, *E2F1* or *YBX1* genes were synthesized by Umine Biotechnology or GenePharma (**Supplementary Table 10**). Transfections with siRNA (50 nM) were performed with lipofectamine 2000 (Life Technologies, Carlsbad, USA). Briefly, ESCC cells were seeded in 6-well plates with complete culture medium without antibiotics one day before transfection. When cells were at appropriate confluent, they were maintained in serum-free culture medium and transfected with siRNA-lipofectamine 2000 mixture. The medium was substituted with complete culture medium at 6 h after transfection. The transfected cells were cultured for 48 h and then were harvested for transfection efficiency validation or other analysis.

Plasmids, lentivirus production and transduction

To achieve depletion of *NSUN2*, *LIN28B* or *GRB2* in cells, short hairpin RNA (shRNA) specifically targeting *NSUN2*, *LIN28B* or *GRB2* (**Supplementary Table 10**) was respectively synthesized and inserted into pLKD-CMV-Puro-U6 (Obio Technology, Shanghai, China), pLKD-U6-MCS-CMV-Puro (Umine Biotechnology Co.,LTD, Guangzhou, China) or pLKD-U6-MCS-CMV-Blasticidin (Umine Biotechnology Co.,LTD, Guangzhou, China) lentiviral shRNA vector. Plasmids containing shNSUN2-insensitive wild-type (WT) and catalytic mutant *NSUN2* were constructed as described in previous studies [4,9]. One mutant (MUT1) carried a point mutation at catalytic site (C321A) while another mutant (MUT2) carried point mutations at both catalytic site (C321A) and releasing site (C271A). Full-length cDNA of shNSUN2-insensitive wild-type and catalytic mutant *NSUN2* was synthesized and subcloned into pLenti-CMV-MCS-PGK-Puro-WPRE lentiviral expression vector (Obio Technology, Shanghai, China) to construct lentiviral vector

expressing wild-type or catalytic mutant *NSUN2*. For the *GRB2* overexpression vector, synthesized *GRB2* sequence was inserted into pLVX-EF1a-Puro-WPRE-CMV-MCS vector (Umine Biotechnology Co.,LTD, Guangzhou, China). HEK293T cells were seeded with complete culture medium without antibiotics one day before transfection. When the cells were at proper confluent, they were transfected with the vector described above and the lentiviral vector packaging system (Obio Technology, Shanghai, China) using lipofectamine 2000 to produce lentiviruses. The lentiviral supernatant was harvested at 48 and 72 h post-transduction and filtered through 0.45-μm PVDF filters. Then the lentiviruses were concentrated by centrifugation and dissolved in the DMEM medium. ESCC cells were infected with the resultant lentiviruses in the presence of polybrene (Sigma-Aldrich, St. Louis, MO, USA) and were maintained in complete culture medium 24 h after infection, followed by selection with puromycin (2 μg/ml) or blasticidin (5 μg/ml) for two weeks. Transfection efficiency was confirmed by western blotting. The corresponding scrambled shRNA vectors or empty lentiviral vectors were used as negative controls.

Construction of vectors

To construct expression vector for FLAG-tagged LIN28B, synthesized cDNA encoding full-length (LIN28B-WT) or CSD domain (residues 21−112) truncated (LIN28B-ΔCSD) or W36A mutant of LIN28B were subcloned into pcDNA3.1-3× FLAG vector (Umine Biotechnology Co.,LTD, Guangzhou, China).

RNA stability assay

Cells were seeded in 6-well plates and treated with 5 μg/ml actinomycin D for 8 h, 6 h, 4 h, 2 h and 0 h before cell collection. Total RNA (2 μg) was extracted with TRIzol reagent (Invitrogen,

Carlsbad, CA, USA) and analyzed by qRT-PCR. The mRNA half-life time was calculated as previously described [21].

Luciferase reporter gene assays

For promoter reporter assays, the *NSUN2* promoter sequence (-1000 bp from the transcription start site) with wild-type binding site (TGCGCGCGAAG) of E2F1 and the corresponding oligos with mutant binding site (GGGATTCTTTG) of E2F1 were designed, synthesized and cloned into pGL4-promoter vector by Umine Biotechnology (Guangzhou, China). ESCC cells were seeded in 24-well plates and were co-transfected with 500 ng pGL4-promoter vectors, 100 ng pRL-SV40 Renilla vector (Promega, Madison, WI, USA) and 50 nM siRNA targeting *E2F1* mRNA or scramble. The transfected cells were cultured for 48 h and harvested for luciferase activity detection by Dual-Luciferase Reporter Assay System (Promega, Madison, WI, USA). The relative Fluc/Rluc activity was calculated by normalizing the activity of firefly luciferase to that of renilla luciferase. For *GRB2*-3'UTR reporter assays, *GRB2* 3'UTR with wild-type (GRB2-WT) or cytosine to guanine mutant m^5C site (chr17:75318971; GRB2-MUT) was cloned into the downstream region of pmirGLO vector (Obio Technology, Shanghai, China). 300 ng pmirGLO vectors were co-transfected with 100 ng pRL-SV40 Renilla vector into ESCC cells with or without *NSUN2* or *LIN28B* knockdown in 24-well plates using lipofectamine 2000. The luciferase activity or RNA level was examined 48 h after transfection using the Dual-Luciferase Reporter Assay System or qRT-PCR assays, respectively, and was normalized using Renilla luciferase activity or mRNA level. Primers specific for Firefly luciferase and Renilla luciferase are listed in **Supplementary Table 8**.

RNA pulldown and mass spectrometry analysis

Biotin-labeled RNA fragment (50 pmol) containing 50-bp *GRB2* RNA sequences with (*GRB2*[m^5C]) or without (*GRB2*[C]) m^5C modification at m^5C site (chr17:75318971) were synthesized from Ruibiotech (Beijing, China; **Supplementary Table 10**) and incubated with protein extracts from KYSE30 cells. Streptavidin beads were then added and the bead-bound proteins were extracted and subjected to mass spectrometry or western blotting analysis. To identify proteins preferentially binding to *GRB2*[m^5C] RNA, we used the following criteria: unique peptides > 10, ratio of average LFQ intensity of *GRB2*[m^5C] and *GRB2*[C] > 2.

Protein expression and purification

HEK293T cells were transfected with Flag-tagged wild-type or mutant (CSD truncated or W36A) *LIN28B* plasmids using lipofectamine 2000. The transfected cells were cultured for 48 h, and were harvested and lysed with lysis buffer followed by sonication at 4 °C. The lysate was then centrifuged at 12 000 rpm for 10 min and the clear lysate was incubated with Anti-Flag Affinity Gel (KAP0064, Dia-An Biotech, Wuhan, China) at 4 °C overnight. Then the bound complex was incubated with 3× Flag peptide elution buffer to elute the bound proteins. The purified proteins were verified with SDS-PAGE followed by western blotting.

Computational model structure analysis

The RNA structure was modelled from YBX1-m^5C RNA complex (PDBID: 6A6L) [9] through Web 3DNA (w3DNA) 2.0 [22]. Then the LIN28B-m^5C RNA complex structure was modelled through molecular docking by HDOCK [23]. The structure was visualized by PyMol (Version 1.8.6.0). The residue interaction network was analyzed by RING software [24].

RNA electrophoretic mobility shift assays (REMSA)

Assays were performed using the LightShift Chemiluminescent RNA EMSA Kit (Thermo Fisher Scientific, Waltham, MA, USA). Biotin-labeled RNA probes were synthesized by Ruibiotech (Beijing, China; **Supplementary Table 10**). Briefly, 1 µl biotin-labeled RNA probes (4 nM final concentration) were incubated with different concentrations (0−8 µM) of purified full-length or mutant LIN28B proteins in binding buffer (10 mM HEPES pH 7.3, 20 mM KCl, 1 mM MgCl2, 1 mM DTT, 5% glycerol, and 40 U/ml RNasin) at room temperature for 30 min. The RNA-protein mixtures were separated in 8% native polyacrylamide gels at 4 °C for 1 hour. Complexes was then transferred to a nylon transfer membrane, cross-linked to the membrane using the UVP cross-linker and detected by chemiluminescence.

DNA methylation data analysis

The Illumina HM450K methylation data and the matched RNA-seq data of esophageal squamous cell carcinoma (n = 82) were downloaded from the TCGA database. We used the HOMER analysis tool [25] to analyse the methylation of *NSUN2* TSS region (default -1000bp to +100bp from the transcription start site of *NSUN2*). According to HOMER, a total of 13 probes annotated to *NSUN2* TSS region were included in the methylation analysis. The methylation level of each probe was measured as β value. We used the average β value of the 13 probes to represent the methylation level of *NSUN2* TSS region. Then Spearman's correlation analysis was used to examine the correlation between DNA methylation β value of *NSUN2* TSS region and *NSUN2* gene expression (FPKM) value.

Public data processing

To investigate the differential expression of indicated genes, a public microarray dataset of 179 paired ESCC (GSE53625) [26] was downloaded from the Gene Expression Omnibus. The

differential expression levels of indicated genes were compared by two-sided paired *t* test. Prediction of TFs binding sites in *NSUN2* gene promoter region (-1,000 bp to transcription start site) were performed using JASPAR (PMID: 31701148), AnimalTFDB (PMID: 30204897), ChIPBase (PMID: 27924033), GTRD (PMID: 30445619) and hTFtarget (PMID: 32858223) and TFs-*NSUN2* co-expression analysis was integrated. Only TFs occurred in all these five databases and with positive correlation coefficient r > 0.30 were considered, thus resulting in 4 potential TFs including TFAP2C, SP1, NRF1 and E2F1. Co-expressions of TFs and *NSUN2* were evaluated with Spearman's correlation analysis based on the GSE53625 dataset. Correlations between RNA levels and DNA methylation status of *NSUN2* were analyzed with Spearman's correlation analysis using data of ESCC from The Cancer Genome Atlas Program (TCGA). Copy number alterations (CNV) or mutations of *NSUN2* DNA were analyzed using data of ESCC from International Cancer Genome Consortium (ICGC), University of California at Los Angeles (UCLA) and the TCGA program.

Statistical analysis

Data in our work are presented as the mean ± standard error of the mean (SEM) of at least three biological replicates. A nonparametric test was used to assess data with an abnormal distribution, while two-sided Student's *t*-test was performed to compare difference between two means when the data showed a normal distribution. The relationships between clinicopathological characteristics and indicates gene expression or patient death were determined by using two-sided Chi-square test or Fisher's exact tests. Spearman's correlations were used to determine the relationships between two continuous variables, and $P < 0.05$ and $|r| > 0.30$ was considered significant. We used the two-sided log-rank test in univariate

survival analyses and the Cox proportional hazards model in multivariate survival analyses. The Kaplan-Meier plot was used for presentation. The hazard ratio (HR) and 95% confidence interval (CI) were calculated with age, sex, family history, smoking status, drinking status, treatment, tumor differentiation and tumor stage as covariates. We chose median value as cutoff value to distinguish patients with high (≥ median) or low (< median) levels of RNA or m^5C of indicated genes. An IHC score of 6 was chosen as cut-off value for distinguishing patients' high (> 6) or low (≤ 6) expression of indicated protein. All the statistical analyses were performed using SPSS version 20.0 software (SPSS Inc., Chicago, IL, USA) or GraphPad Prism 8.0 software (GraphPad Software, La Jolla, CA, USA). $P < 0.05$ was considered statistically significant.

URLs

ChIPBase v2.0, http://rna.sysu.edu.cn/chipbase/; Gene Transcription Regulation Database (GTRD), http://gtrd.biouml.org/; Database of Human Transcription Factors Targets (hTFtarget), http://bioinfo.life.hust.edu.cn/hTFtarget; Animal Transcription Factor Database (AnimalTFDB), http://bioinfo.life.hust.edu.cn/AnimalTFDB/; JASPAR, http://jaspar.genereg.net/; GENCODE database, https://www.gencodegenes.org/; The Gene Expression Omnibus, https://www.ncbi.nlm.nih.gov/geo/; Integrative Genomics Viewer (IGV), http://www.igv.org/.

Data availability

The accession number for the RNA-BisSeq, RNA-Seq and LIN28B PAR-CLIP-Seq data reported in this paper is HRA000293 (https://bigd.big.ac.cn/gsa-human/browse/HRA000293).

References

1. Sobin LH, Compton CC. TNM seventh edition: what's new, what's changed: communication from the International Union Against Cancer and the American Joint Committee on Cancer. *Cancer* 2010; **116**: 5336−5339.
2. Schneeberger C, Speiser P, Kury F, Zeillinger R. Quantitative detection of reverse transcriptase-PCR products by means of a novel and sensitive DNA stain. *PCR Methods Appl* 1995; **4**: 234−238.
3. Tang XH, Knudsen B, Bemis D, Tickoo S, Gudas LJ. Oral cavity and esophageal carcinogenesis modeled in carcinogen-treated mice. *Clin Cancer Res* 2004; **10**: 301−313.
4. Yang X, Yang Y, Sun BF, Chen YS, Xu JW, Lai WY *et al.* 5-methylcytosine promotes mRNA export - NSUN2 as the methyltransferase and ALYREF as an m(5)C reader. *Cell Res* 2017; **27**: 606−625.
5. Chen S, Zhou Y, Chen Y, Gu J. fastp: an ultra-fast all-in-one FASTQ preprocessor. *Bioinformatics* 2018; **34**: i884−i890.
6. Rieder D, Amort T, Kugler E, Lusser A, Trajanoski Z. meRanTK: methylated RNA analysis ToolKit. *Bioinformatics* 2016; **32**: 782−785.
7. Quinlan AR, Hall IM. BEDTools: a flexible suite of utilities for comparing genomic features. *Bioinformatics* 2010; **26**: 841−842.
8. Harrow J, Frankish A, Gonzalez JM, Tapanari E, Diekhans M, Kokocinski F *et al.* GENCODE: the reference human genome annotation for The ENCODE Project. *Genome Res* 2012; **22**: 1760−1774.
9. Chen X, Li A, Sun BF, Yang Y, Han YN, Yuan X *et al.* 5-methylcytosine promotes pathogenesis of bladder cancer through stabilizing mRNAs. *Nat Cell Biol* 2019; **21**:

978−990.

10. Kim D, Langmead B, Salzberg SL. HISAT: a fast spliced aligner with low memory requirements. *Nat Methods* 2015; **12**: 357−360.

11. Anders S, Pyl P, Huber W. HTSeq—A Python framework to work with high-throughput sequencing data. *Bioinformatics* 2014; **31**: 166−169.

12. Anders S, Huber W. Differential expression analysis for sequence count data. *Genome Biol* 2010; **11**: R106.

13. Veschi V, Liu Z, Voss T, Ozbun L, Gryder B, Yan C *et al.* Epigenetic siRNA and Chemical Screens Identify SETD8 Inhibition as a Therapeutic Strategy for p53 Activation in High-Risk Neuroblastoma. *Cancer Cell* 2017; **31**: 50−63.

14. Yang Y, Wang L, Han X, Yang WL, Zhang M, Ma HL *et al.* RNA 5-Methylcytosine Facilitates the Maternal-to-Zygotic Transition by Preventing Maternal mRNA Decay. *Mol Cell* 2019; **75**: 1188−1202 e1111.

15. Hafner M, Landthaler M, Burger L, Khorshid M, Hausser J, Berninger P *et al.* Transcriptome-wide identification of RNA-binding protein and microRNA target sites by PAR-CLIP. *Cell* 2010; **141**: 129−141.

16. Hafner M, Landthaler M, Burger L, Khorshid M, Hausser J, Berninger P *et al.* PAR-CliP--a method to identify transcriptome-wide the binding sites of RNA binding proteins. *J Vis Exp* 2010; **41**: e2034.

17. Li A, Chen YS, Ping XL, Yang X, Xiao W, Yang Y *et al.* Cytoplasmic m(6)A reader YTHDF3 promotes mRNA translation. *Cell Res* 2017; **27**: 444−447.

18. Langmead B, Trapnell C, Pop M, Salzberg SL. Ultrafast and memory-efficient alignment

of short DNA sequences to the human genome. *Genome Biol* 2009; **10**: R25.

19. Corcoran DL, Georgiev S, Mukherjee N, Gottwein E, Skalsky RL, Keene JD *et al.* PARalyzer: definition of RNA binding sites from PAR-CLIP short-read sequence data. *Genome Biol* 2011; **12**: R79.

20. Robinson J, Thorvaldsdóttir H, Winckler W, Guttman M, Lander E, Getz G *et al.* Integrated Genomics Viewer. *Nat biotechnol* 2011; **29**: 24−26.

21. Huang H, Weng H, Sun W, Qin X, Shi H, Wu H *et al.* Recognition of RNA N(6)-methyladenosine by IGF2BP proteins enhances mRNA stability and translation. *Nat Cell Biol* 2018; **20**: 285−295.

22. Li S, Olson WK, Lu XJ. Web 3DNA 2.0 for the analysis, visualization, and modeling of 3D nucleic acid structures. *Nucleic Acids Res* 2019; **47**: W26−W34.

23. Yan Y, Zhang D, Zhou P, Li B, Huang SY. HDOCK: a web server for protein-protein and protein-DNA/RNA docking based on a hybrid strategy. *Nucleic Acids Res* 2017; **45**: W365−W373.

24. Piovesan D, Minervini G, Tosatto SC. The RING 2.0 web server for high quality residue interaction networks. *Nucleic Acids Res* 2016; **44**: W367−374.

25. Heinz S, Benner C, Spann N, Bertolino E, Lin YC, Laslo P *et al.* Simple combinations of lineage-determining transcription factors prime cis-regulatory elements required for macrophage and B cell identities. *Mol Cell* 2010; **38**: 576−589.

26. Li J, Chen Z, Tian L, Zhou C, He MY, Gao Y *et al.* LncRNA profile study reveals a three-lncRNA signature associated with the survival of patients with oesophageal squamous cell carcinoma. *Gut* 2014; **63**: 1700−1710.

Su et al. Supplementary Figure 1

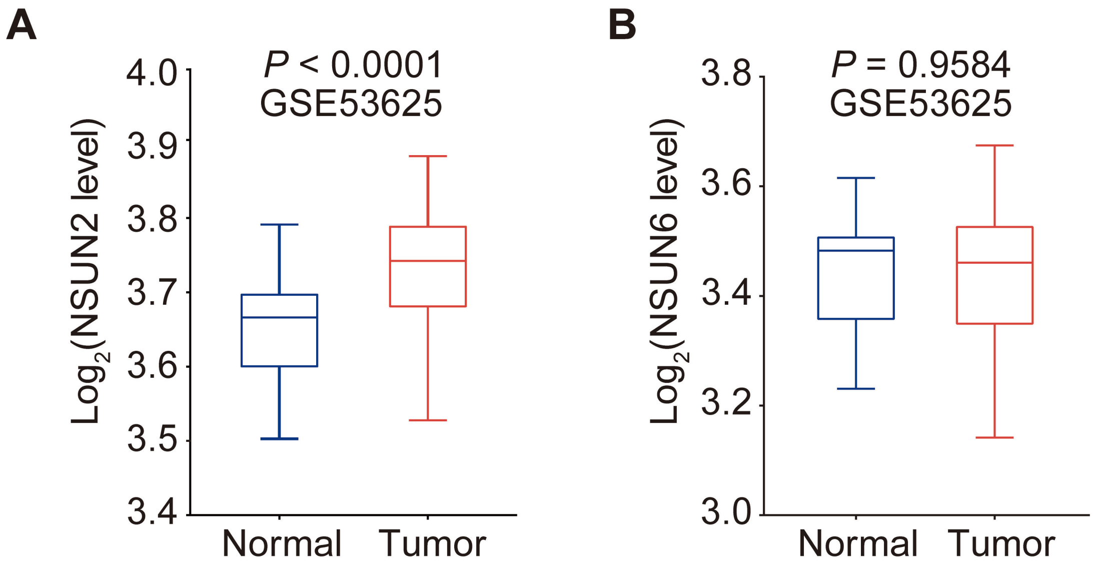

Supplementary Fig. 1 Expression levels of *NSUN2* and *NSUN6* in patients with ESCC. Related to Fig. 1.

(**A**–**B**) RNA levels of *NSUN2* (**A**) but not *NSUN6* (**B**) were significantly higher in ESCC tumors than in paired normal tissues from a public microarray dataset (GSE53625) of 179 paired ESCC cohort. Data are represented as boxplots. The centerlines of the box represent median, while the upper and lower hinges indicate 25th and 75th percentiles, respectively. *P*-values were calculated by two-sided paired *t* test.

Su et al. Supplementary Figure 2

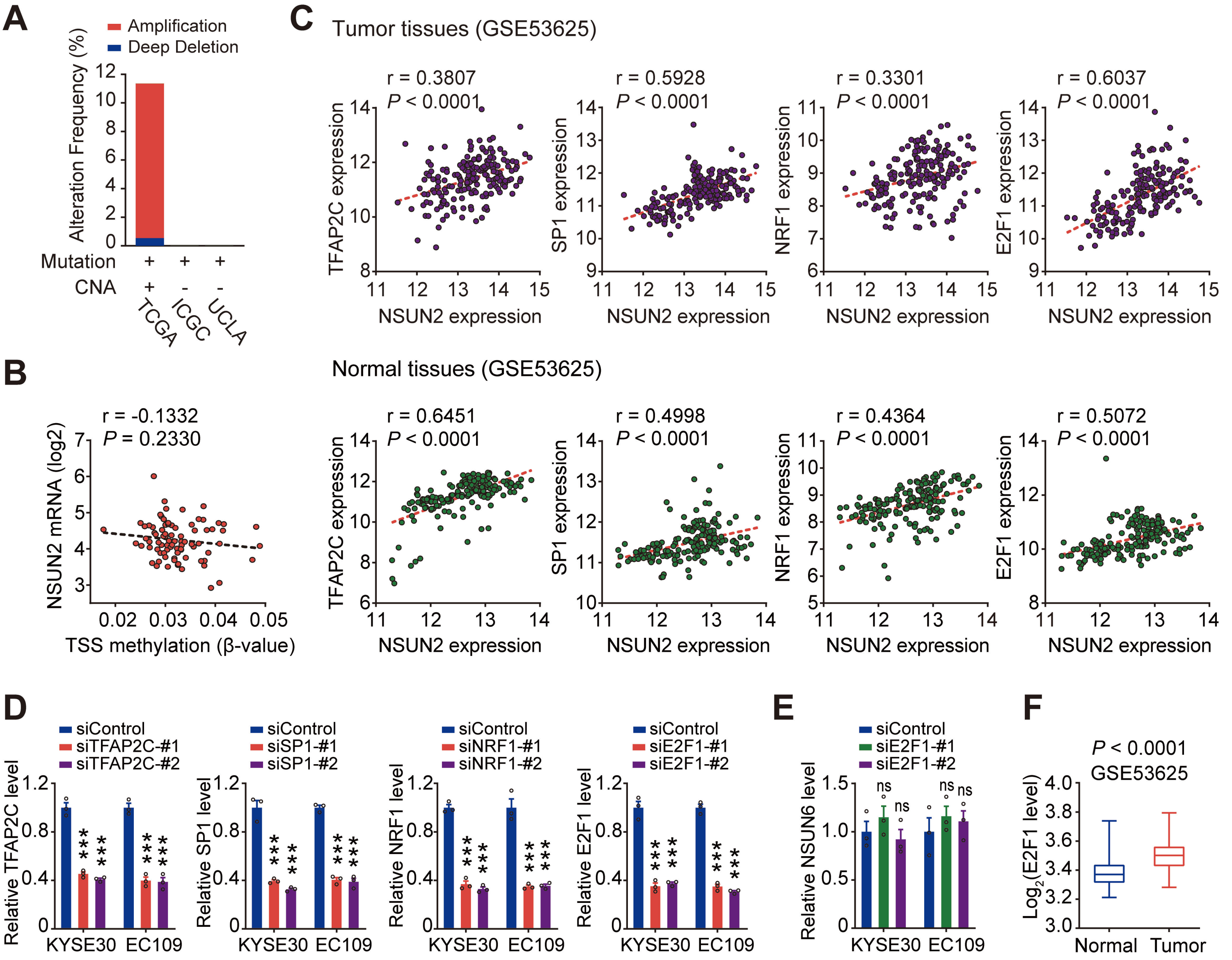

Supplementary Fig. 2 Correlation between expressions of potential transcription factors and *NSUN2* at mRNA levels. Related to Fig. 2.

(**A**) Copy number or mutation of *NSUN2*. Both *NSUN2* mutation and amplification are null or very rare in patients reported in International Cancer Genome Consortium (ICGC), University of California at Los Angeles (UCLA) and the TCGA project. (**B**) No correlation between *NSUN2* gene methylation status and mRNA levels from the TCGA ESCC data (n = 82). (**C**) Spearman's correlations between expressions of suggested transcription factors and *NSUN2* at mRNA levels in a public microarray dataset (GSE53625) of ESCC tumors (n = 179, upper panel) or non-tumor tissues (n = 179, lower panel). (**D**) siRNA silencing efficiencies of *TFAP2C*, *SP1*, *NRF1*, or *E2F1* expression. (**E**) Relative *NSUN6* RNA levels in ESCC cells with or without knockdown of *E2F1*. (**F**) *E2F1* RNA levels were significantly higher in ESCC tumors than in paired normal tissues from a public microarray dataset (GSE53625) of 179 paired ESCC cohort. Results in (**D**−**E**) are mean ± SEM from 3 independent experiments. Data represent as boxplots in (**F**); the centerline represents the median, while the upper and lower hinges indicate the 25th and 75th percentiles, respectively. P-values were calculated using two-sided Student's t test in (**D**−**E**) and two-sided paired t test in (**F**). *P < 0.05, **P < 0.01, ***P < 0.001, ns, not significant.

Su et al. Supplementary Figure 3

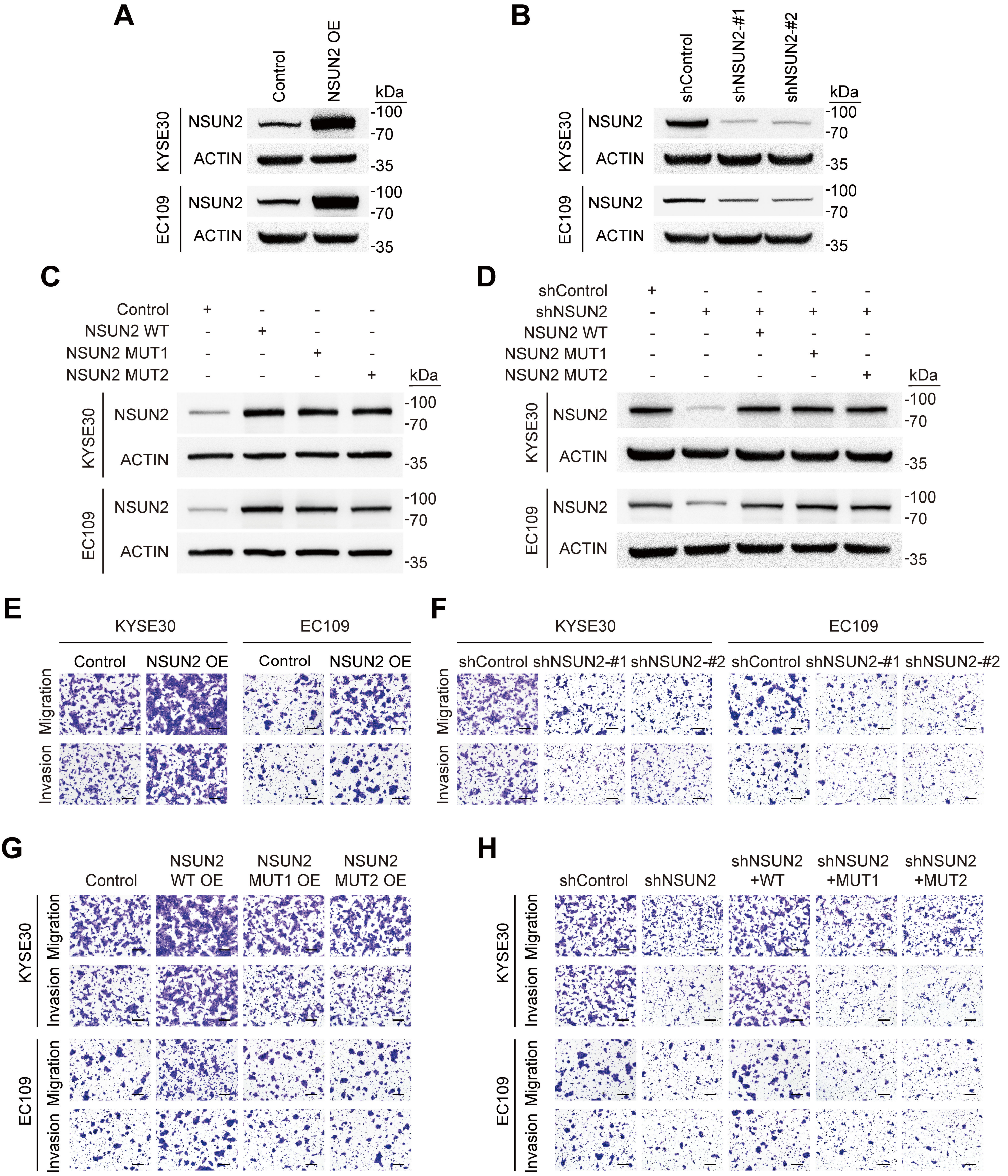

Supplementary Fig. 3 Effects of NSUN2 on ESCC cell migration and invasion. Related to Fig. 3. (**A**−**D**) Efficiency of *NSUN2* overexpression (**A**), knockdown (**B**), wild-type or mutant *NSUN2* overexpression (**C**) in ESCC cells, and *NSUN2*-depleted cells rescued with overexpression of wild-type or mutant *NSUN2* (**D**). ACTIN served as a control. (**E**−**F**) Representative pictures showing the effects of *NSUN2* overexpression (**E**) or knockdown (**F**) on migration and invasion of ESCC cells. (**G**−**H**) Representative images indicating that wild-type but not mutant *NSUN2* enhanced migration and invasion of ESCC cells (**G**) and reversed the inhibition of migration and invasion caused by *NSUN2* knockdown (**H**). Scale bars, 200 μm. Images were photographed from 3 random fields. WT, wild-type *NSUN2* plasmids; MUT1, *NSUN2* plasmids with a point mutation at catalytic site (C321A); MUT2, *NSUN2* plasmids with point mutations at both catalytic site (C321A) and releasing site (C271A), respectively. All three plasmids were insensitive to shNSUN2 plasmid. All data are from 3 independent experiments.

Su et al. Supplementary Figure 4

A

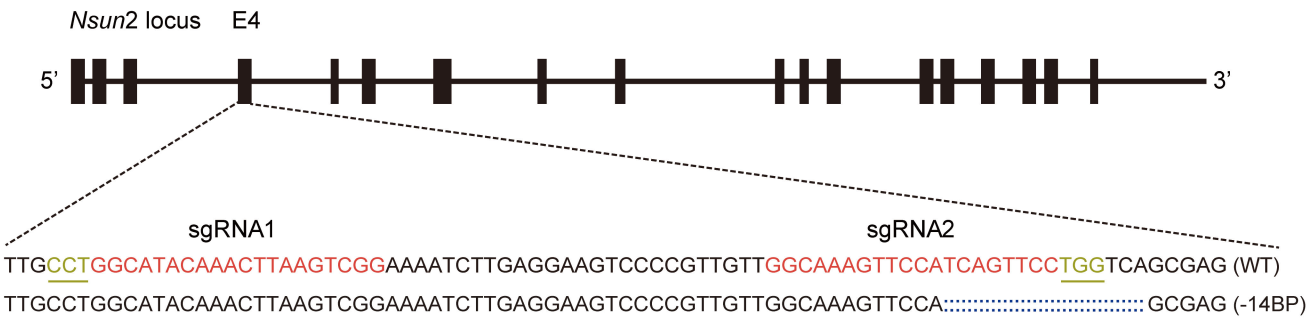

B

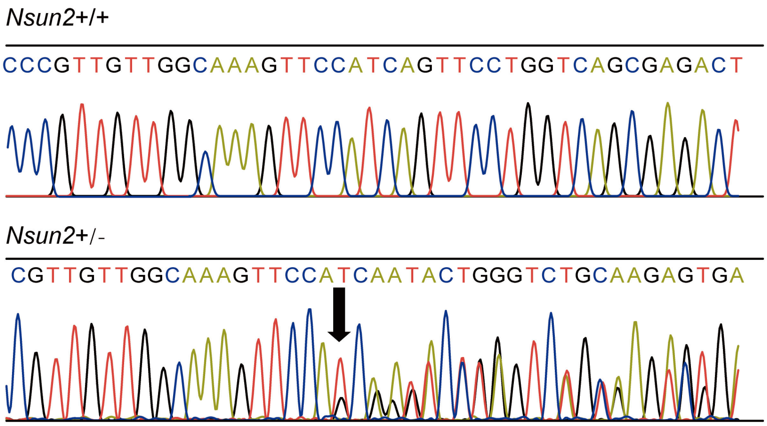

C

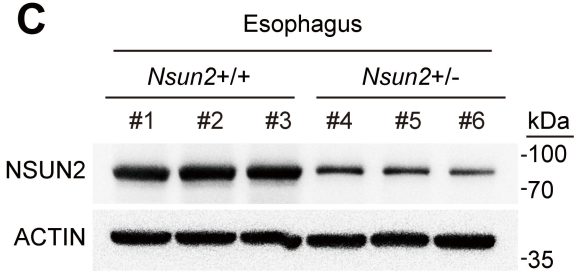

D

	Nsun2+/+	*Nsun2+/-*
Atypical hyperplasia	0	3
Carcinoma in situ	1	6
Invasive carcinoma	9	1

Supplementary Fig. 4 Validation of *Nsun2* knockout mice. Related to Fig. 3.
(**A**) Schematic diagram of sgRNAs targeting the *Nsun2* locus. One *Nsun2* allele was disrupted by injecting Cas9 mRNAs and two small guide RNAs targeting the fourth exon of the gene into fertilized eggs. PAM sequences are underlined and highlighted in yellow. sgRNA targeting sites are in red. The 14-bp shift due to the deletion mutation is in blue. (**B**) Sanger sequencing of mouse tail DNA PCR amplification products confirming the 14-bp shift due to the deletion mutation of *Nsun2*. The deleted nucleotides are indicated with black arrow. (**C**) Western blotting analysis of esophageal tissues from *Nsun2*+/+ and *Nsun2*+/- mice confirming the decreased expression of NSUN2 protein. Analysis was performed once with three mice per genotype. ACTIN was used as a control. (**D**) Number of mice with different pathological degree of esophageal masses in *Nsun2*+/+ mice and their *Nsun2*+/- littermates after 4-NQO withdrawal for 12 weeks.

Su et al. Supplementary Figure 5

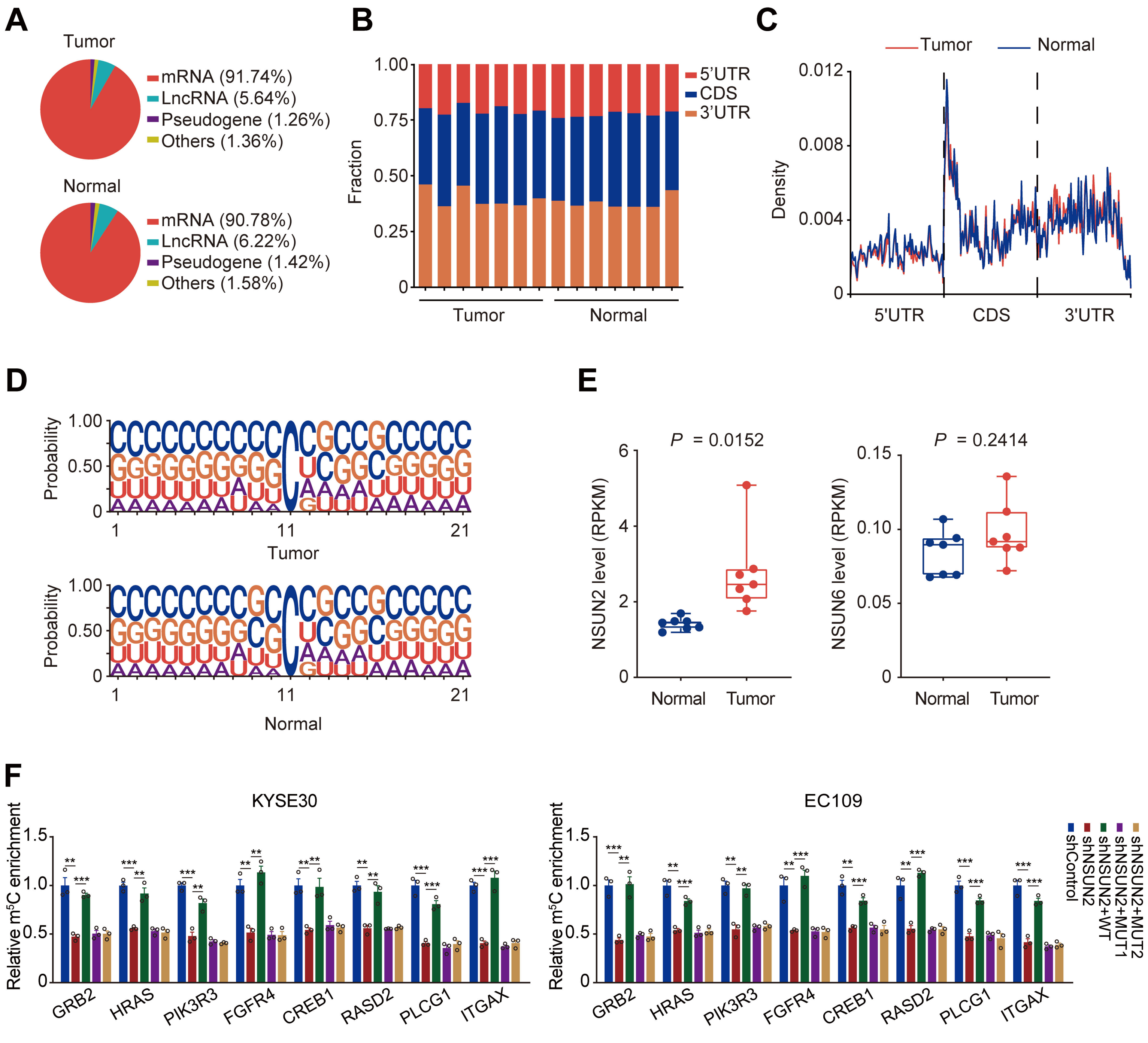

Supplementary Fig. 5 Distribution profiles of RNA m^5C modification in human ESCC. Related to Fig. 4.

(**A**) The proportion of RNA categories with m^5C sites in ESCC tumors and paired normal samples. (**B**) Transcriptome-wide distribution of mRNA m^5C sites in the CDS, 5′UTR and 3′UTR of mRNA transcripts in ESCC tumors and paired normal samples. (**C**) Metagene profiles showing the m^5C methylation density along transcripts identified in ESCC tumors and paired normal samples. (**D**) Sequence context of m^5C sites identified in ESCC tumors and paired normal samples. (**E**) Aberrant overexpression of *NSUN2* but not *NSUN6* RNA in ESCC tumors than in paired normal tissues by RNA-Seq (n = 7). (**F**) Wild-type but not mutant *NSUN2* reversed the decreased m^5C levels of genes involved in cancer-related pathways caused by *NSUN2* depletion using m^5C-RIP-qPCR from 3 independent experiments. P-values are calculated by two-sided paired t test in (**E**) and by two-sided Student's t test in (**F**) (*$P < 0.05$, **$P < 0.01$ and ***$P < 0.001$).

Su et al. Supplementary Figure 6

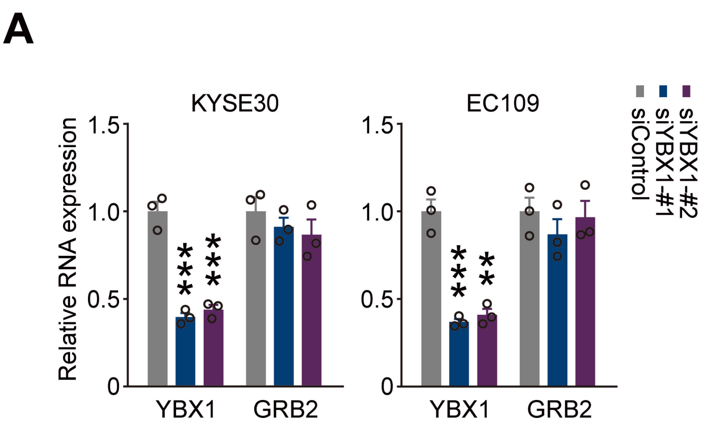

B

Protein ID	Gene Name	Ratio (m^5C/C)
Q6ZN17	LIN28B	80.806760
P14625	HSP90B1	6.852360
P14618	PKM	3.779213
O75534	CSDE1	2.797942
P06733	ENO1	2.140714
Q99459	CDC5L	2.035171
Q08AE8	SPIRE1	2.024218

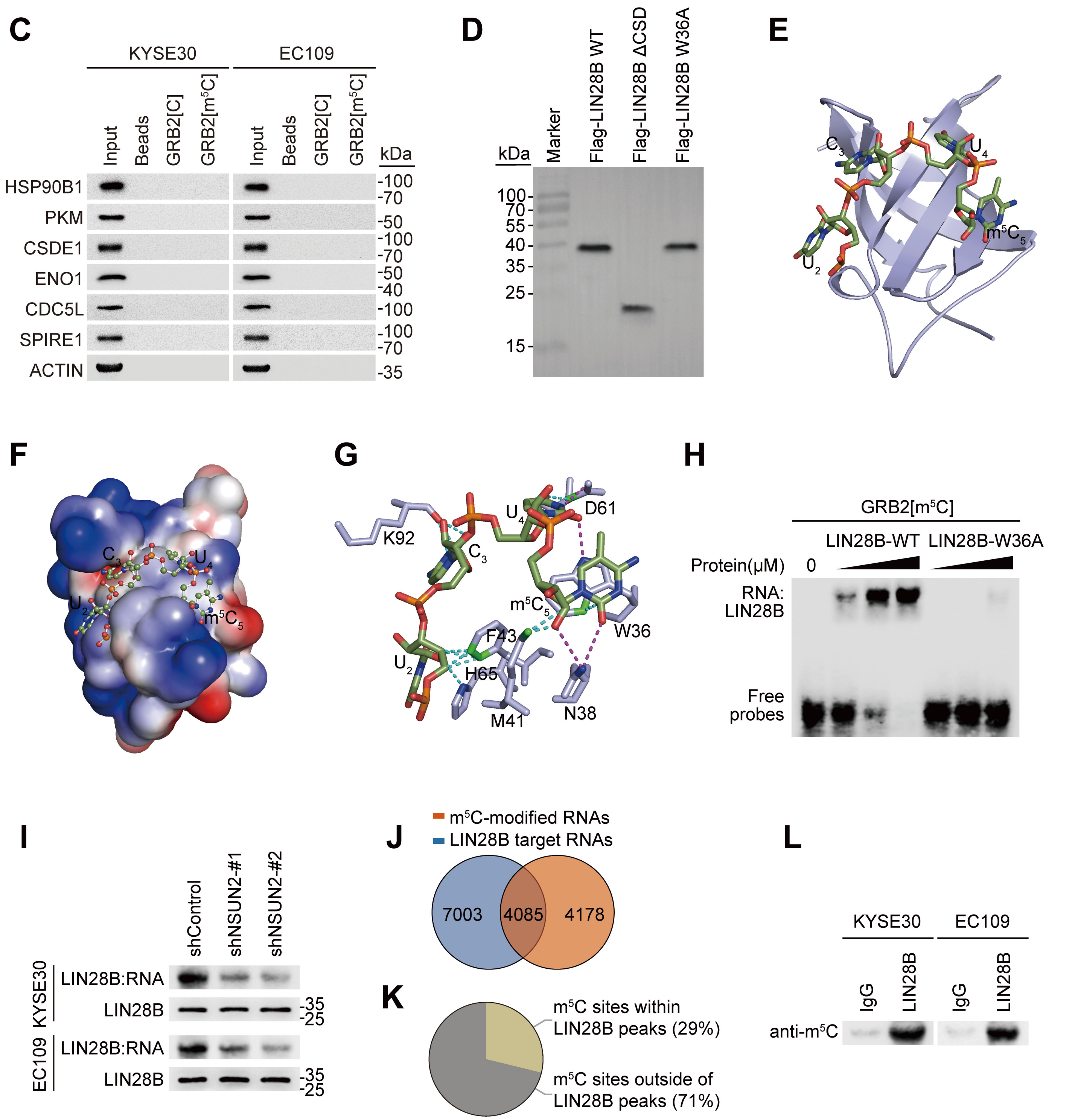

Supplementary Fig. 6 Validation of the binding abilities of potential *GRB2*[m^{5}C] binding proteins to methylated or unmethylated *GRB2* probes. Related to Fig. 6.

(**A**) *YBX1* silencing efficiency and its effect on *GRB2* expression by qRT-PCR from 3 independent experiments. *P*-values are calculated by two-sided Student's *t* test (*$P < 0.05$, **$P < 0.01$ and ***$P < 0.001$). (**B**) List of proteins with higher *GRB2*[m^{5}C] binding affinity identified by RNA pulldown followed by mass spectrometry analysis. The filter criteria are unique peptides > 10, ratio of average LFQ intensity of *GRB2*[m^{5}C] and *GRB2*[C] > 2. (**C**) Western blotting analysis of potential *GRB2*[m^{5}C] binding proteins obtained from RNA pulldown followed by mass spectrometry analysis. (**D**) Western blotting showing the Flag-tagged wild-type (WT) and mutant (CSD truncated or W36A) LIN28B proteins. Positions of molecular markers are indicated on the left panel. (**E**) An overall view of the LIN28B CSD domain in complex with the *GRB2* m^{5}C RNA oligo. The LIN28B CSD domain is displayed as a purple ribbon, and the *GRB2* m^{5}C RNA oligo is shown as a stick model. (**F**) Electrostatic potential of the surface of LIN28B CSD in complex with the *GRB2* m^{5}C RNA oligo. (**G**) Recognition of m^{5}C by the LIN28B CSD domain. (**H**) REMSA assays of *GRB2*[m^{5}C] probes with purified FLAG-tagged LIN28B (wild-type or W36A mutants). (**I**) PAR-CLIP assays of LIN28B protein in control and *NSUN2*-deficient ESCC cells. The LIN28B pull-down RNAs were labelled with biotin and visualized by the chemiluminescent nucleic acid detection module. (**J**–**K**) Overlay of LIN28B-binding RNAs with m^{5}C-modified RNAs (**J**) and distributions of m^{5}C sites within and outside of LIN28B binding peaks (**K**) as determined by LIN28B PAR-CLIP-Seq and RNA-BisSeq. (**L**) PAR-CLIP assays of m^{5}C-modified RNAs pulled down by LIN28B in ESCC cells. The LIN28B-bound RNAs were incubated with anti-m^{5}C antibody and visualized by chemiluminescent assays.

Su et al. Supplementary Figure 7

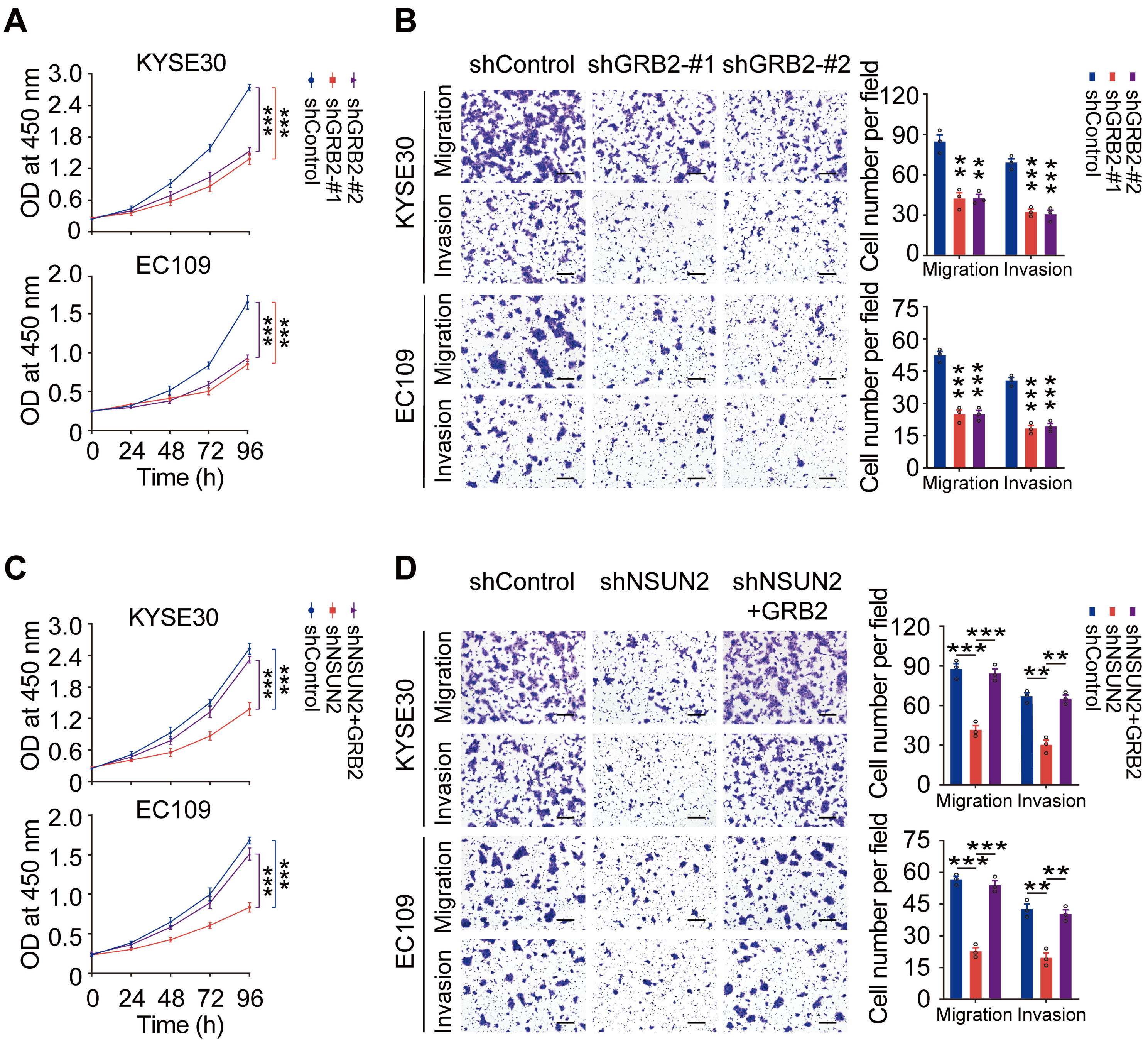

Supplementary Fig. 7 Effects of NSUN2-GRB2 axis on malignant cell phenotypes of ESCC cells. Related to Fig. 7.

(**A**−**B**) Effects of *GRB2* knockdown on abilities of ESCC cell proliferation (**A**), migration and invasion (**B**). (**C**−**D**) Overexpression of *GRB2* significantly reversed the inhibitory effects of *NSUN2* knockdown on ESCC cell proliferation (**C**), migration and invasion (**D**). The left panel shows representative images of cell migration and invasion, and the right panel shows quantitative statistics in (**B**) and (**D**). All results represent as mean ± SEM of at least three independent experiments. Images were photographed from 3 random fields. *$P < 0.05$, **$P < 0.01$ and ***$P < 0.001$ of two-sided Student's t test.

Supplementary Table 1. Baseline demographic and clinical characteristics of individuals with ESCC used for qRT-PCR and m^5C-RIP-qPCR in this study.

**Variable**	**All cases (*N* = 215)**	**Alive (*N* = 93)**	**Deceased (*N* = 122)**	***P* value[d]**
Age(years), mean (S.E.M.[a])	60.09 (0.62)	59.86 (0.88)	60.27 (0.87)	0.744
Sex, *N* (%)				0.115
Male	166 (77.2)	67 (72.0)	99 (81.1)	
Female	49 (22.8)	26 (28.0)	23 (18.9)	
Family history, *N* (%)				0.631
Yes	52 (24.2)	21 (22.6)	31 (25.4)	
No	163 (75.8)	72 (77.4)	91 (74.6)	
Smoking status[b], *N* (%)				0.351
Ever	137 (63.7)	56 (60.2)	81 (66.4)	
Never	78 (36.3)	37 (39.8)	41 (33.6)	
Drinking status[b], *N* (%)				0.460
Ever	94 (43.7)	38 (40.9)	56 (45.9)	
Never	121 (56.3)	55 (59.1)	66 (54.1)	
Differentiation, *N* (%)				0.858
Well	37 (17.2)	17 (18.3)	20 (16.4)	
Moderate	114 (53.0)	50 (53.8)	64 (52.5)	
Poor	64 (29.8)	26 (27.9)	38 (31.1)	
Tumor stage[c], *N* (%)				< 0.0001
I	13 (6.0)	8 (8.6)	5 (4.1)	
II	93 (43.3)	54 (58.1)	39 (32.0)	
III	101 (47.0)	28 (30.1)	73 (59.8)	
IV	8 (3.7)	3 (3.2)	5 (4.1)	
Treatment, *N* (%)				0.283
Surgery Only	203 (94.4)	87 (93.5)	116 (95.1)	
Surgery + Chemotherapy	5 (2.3)	3 (3.2)	2 (1.6)	
Surgery + Radiotherapy	2 (1.0)	2 (2.2)	0 (0.0)	
Surgery + Chemoradiotherapy	5 (2.3)	1 (1.1)	4 (3.3)	

[a]S.E.M., standard error of mean.

[b]Individuals who smoked an average of <1 cigarette/day and for <1 year in their lifetime were defined as nonsmokers; otherwise, they were defined as smokers. Individuals were classified as drinkers if they drank at least twice a week and continuously for at least 1 year during their lifetime; otherwise, they were defined as nondrinkers.

[c]Tumor staging were reviewed by at least 3 pathologists and defined according to the American Joint Committee on Cancer (AJCC) 7th edition.

[d]*P* value was calculated by two-sided Chi-square test.

Supplementary Table 2. Associations between *NSUN2*, *GRB2* RNA level or *GRB2* m^5C level and clinical characteristics of individuals with ESCC used for qRT-PCR and m^5C-RIP-qPCR in this study.

Variable	All cases (*N* = 215)	Low NSUN2 RNA level[d] (*N* = 107)	High NSUN2 RNA level[d] (*N* = 108)	*P* value[e]	Low GRB2 RNA level[d] (*N* = 107)	High GRB2 RNA level[d] (*N* = 108)	*P* value[e]	Low GRB2 m^5C level[d] (*N* = 107)	High GRB2 m^5C level[d] (*N* = 108)	*P* value[e]
Age(years), mean (S.E.M.[a])	60.09 (0.62)	59.83 (0.83)	60.35 (0.92)	0.676	60.90 (0.88)	59.3 (0.87)	0.197	60.51 (0.81)	59.68 (0.94)	0.500
Sex, *N* (%)				0.395			0.134			0.134
Male	166 (77.2)	80 (74.8)	86 (79.6)		78 (72.9)	88 (81.5)		78 (72.9)	88 (81.5)	
Female	49 (22.8)	27 (25.2)	22 (20.4)		29 (27.1)	20 (18.5)		29 (27.1)	20 (18.5)	
Family history, *N* (%)				0.320			0.120			0.359
Yes	52 (24.2)	29 (27.1)	23 (21.3)		21 (19.6)	31 (28.7)		23 (21.5)	29 (26.9)	
No	163 (75.8)	78 (72.9)	85 (78.7)		86 (80.4)	77 (71.3)		84 (78.5)	79 (73.1)	
Smoking status[b], *N* (%)				0.738			0.079			0.367
Ever	137 (63.7)	67 (62.6)	70 (64.8)		62 (57.9)	75 (69.4)		65 (60.7)	72 (66.7)	
Never	78 (36.3)	40 (37.4)	38 (35.2)		45 (42.1)	33 (30.6)		42 (39.3)	36 (33.3)	
Drinking status[b], *N* (%)				0.444			0.624			0.952
Ever	94 (43.7)	44 (41.1)	50 (46.3)		45 (42.1)	49 (45.4)		47 (43.9)	47 (43.5)	
Never	121 (56.3)	63 (58.9)	58 (53.7)		62 (57.9)	59 (54.6)		60 (56.1)	61 (56.5)	
Differentiation, *N* (%)				0.506			0.802			0.857
Well	37 (17.2)	17 (15.9)	20 (18.5)		17 (15.9)	20 (18.5)		19 (17.8)	18 (16.7)	
Moderate	114 (53.0)	61 (57.0)	53 (49.1)		59 (55.1)	55 (50.9)		58 (54.2)	56 (51.9)	
Poor	64 (29.8)	29 (27.1)	35 (32.4)		31 (29.0)	33 (30.6)		30 (28.0)	34 (31.4)	
Tumor stage[c], *N* (%)				0.006			0.019			0.018
I	13 (6.0)	9 (8.4)	4 (3.7)		11 (10.3)	2 (1.9)		9 (8.4)	4 (3.7)	
II	93 (43.3)	56 (52.3)	37 (34.3)		50 (46.7)	43 (39.8)		55 (51.4)	38 (35.2)	
III	101 (47.0)	40 (37.4)	61 (56.5)		42 (39.3)	59 (54.6)		40 (37.4)	61 (56.5)	
IV	8 (3.7)	2 (1.9)	6 (5.5)		4 (3.7)	4 (3.7)		3 (2.8)	5 (4.6)	
Treatment, *N* (%)				0.197			0.477			0.800
Surgery Only	203 (94.4)	98 (91.6)	105 (97.2)		99 (92.6)	104 (96.3)		102 (95.3)	101 (93.5)	

Surgery + Chemotherapy	5 (2.3)	3 (2.8)	2 (1.9)	3 (2.8)	2 (1.9)	3 (2.8)	2 (1.9)
Surgery + Radiotherapy	2 (1.0)	2 (1.9)	0 (0.0)	1 (0.9)	1 (0.9)	0 (0.0)	2 (1.9)
Surgery + Chemoradiotherapy	5 (2.3)	4 (3.7)	1 (0.9)	4 (3.7)	1 (0.9)	2 (1.9)	3 (2.7)

[a]S.E.M., standard error of mean.

[b]Individuals who smoked an average of <1 cigarette/day and for <1 year in their lifetime were defined as nonsmokers; otherwise, they were defined as smokers. Individuals were classified as drinkers if they drank at least twice a week and continuously for at least 1 year during their lifetime; otherwise, they were defined as nondrinkers.

[c]Tumor staging were reviewed by at least 3 pathologists and defined according to the American Joint Committee on Cancer (AJCC) 7th edition.

[d]High is defined as ≥ median and low is as < median.

[e]*P* value was calculated by two-sided Chi-square test.

Supplementary Table 3. Univariate and multivariate Cox regression analysis for overall survival in ESCC patients.

**Variable**	**Univariate**			**Multivariate**		
	**HR**	**95% CI**	***P* value**	**HR**[a]	**95% CI**[a]	***P* value**
Gender (male vs. female)	1.34	0.85-2.11	0.202	-	-	-
Age (≥60 y vs. < 60 y)	1.26	0.88-1.79	0.208	-	-	-
Smoking (ever vs. never)	1.20	0.83-1.75	0.335	-	-	-
Drinking (ever vs. never)	1.20	0.84-1.71	0.318	-	-	-
Family history (yes vs. no)	1.00	0.67-1.50	0.983	-	-	-
Tumor stage (III/IV vs. I/II)	2.50	1.72-3.62	< 0.001	-	-	-
Differentiation (moderate/poor vs. well)	1.16	0.72-1.88	0.535	-	-	-
Treatment (surg+chemo/radiotherapy vs. surg only)	0.86	0.38-1.96	0.719	-	-	-
NSUN2 RNA level (high vs. low)	2.17	1.50-3.13	< 0.001	1.94	1.32-2.85	< 0.001
GRB2 RNA level (high vs. low)	1.86	1.29-2.68	< 0.001	1.78	1.22-2.61	0.003
GRB2 RNA m^5C level (high vs. low)	2.00	1.39-2.90	< 0.001	1.70	1.16-2.49	0.007

[a]The HR and 95% CI of NSUN2 RNA, GRB2 RNA and GRB2 m^5C were calculated with adjustments for gender, age, smoking status, drinking status, family history, tumor stage, tumor differentiation and treatment as covariates. HR, hazard ratio; CI, confidence interval.

Supplementary Table 4. Baseline demographic and clinical characteristics of individuals with ESCC used for IHC in this study.

Variable	All cases (N = 59)	Alive (N = 24)	Deceased (N = 35)	P value[d]
Age(years), mean (S.E.M.[a])	62.27 (1.12)	62.17 (1.44)	62.34 (1.63)	0.939
Sex, N (%)				
Male	47 (79.7)	17 (70.8)	30 (85.7)	0.287
Female	12 (20.3)	7 (29.2)	5 (14.3)	
Family history, N (%)				
Yes	13 (22.0)	5 (20.8)	8 (22.9)	0.854
No	46 (78.0)	19 (79.2)	27 (77.1)	
Smoking status[b], N (%)				0.297
Ever	39 (66.1)	14 (58.3)	25 (71.4)	
Never	20 (33.9)	10 (41.7)	10 (28.6)	
Drinking status[b], N (%)				0.821
Ever	26 (44.1)	11 (45.8)	15 (42.9)	
Never	33 (55.9)	13 (54.2)	20 (57.1)	
Differentiation, N (%)				0.951
Well	14 (23.7)	6 (25.0)	8 (22.9)	
Moderate	29 (49.2)	12 (50.0)	17 (48.5)	
Poor	16 (27.1)	6 (25.0)	10 (28.6)	
Tumor stage[c], N (%)				0.122
I	3 (5.1)	2 (8.3)	1 (2.9)	
II	22 (37.3)	12 (50.0)	10 (28.6)	
III	32 (54.2)	9 (37.5)	23 (65.6)	
IV	2 (3.4)	1 (4.2)	1 (2.9)	

[a]S.E.M., standard error of mean.

[b]Individuals who smoked an average of <1 cigarette/day and for <1 year in their lifetime were defined as nonsmokers; otherwise, they were defined as smokers. Individuals were classified as drinkers if they drank at least twice a week and continuously for at least 1 year during their lifetime; otherwise, they were defined as nondrinkers.

[c]Tumor staging were reviewed by at least 3 pathologists and defined according to the American Joint Committee on Cancer (AJCC) 7th edition.

[d]P value was calculated by two-sided Chi-square test.

Supplementary Table 5. Associations between *NSUN2*, *GRB2* protein level and clinical characteristics of individuals with ESCC used for IHC in this study.

**Variable**	**All cases (*N* = 59)**	**Low NSUN2[d] (*N* = 27)**	**High NSUN2[d] (*N* = 32)**	***P* value[e]**	**Low GRB2[d] (*N* = 33)**	**High GRB2[d] (*N* = 26)**	***P* value[e]**
Age(years), mean (S.E.M.[a])	62.27 (1.12)	62.44 (1.62)	62.13 (1.58)	0.889	62.30 (1.29)	62.23 (1.98)	0.975
Sex, *N* (%)				0.741			0.851
Male	47 (79.7)	21 (77.8)	26 (81.2)		26 (78.8)	21 (80.8)	
Female	12 (20.3)	6 (22.2)	6 (18.8)		7 (21.2)	5 (19.2)	
Family history, *N* (%)				0.550			0.645
Yes	13 (22.0)	5 (18.5)	8 (25.0)		8 (24.2)	5 (19.2)	
No	46 (78.0)	22 (81.5)	24 (75.0)		25 (75.8)	21 (80.8)	
Smoking status[b], *N* (%)				0.933			0.511
Ever	39 (66.1)	18 (66.7)	21 (65.6)		23 (69.7)	16 (61.5)	
Never	20 (33.9)	9 (33.3)	11 (34.4)		10 (30.3)	10 (38.5)	
Drinking status[b], *N* (%)				0.562			0.809
Ever	26 (44.1)	13 (48.1)	13 (40.6)		15 (45.5)	11 (42.3)	
Never	33 (55.9)	14 (51.9)	19 (59.4)		18 (54.5)	15 (57.7)	
Differentiation, *N* (%)				0.087			0.505
Well	14 (23.7)	10 (37.1)	4 (12.5)		9 (27.3)	5 (19.2)	
Moderate	29 (49.2)	11 (40.7)	18 (56.3)		14 (42.4)	15 (57.7)	
Poor	16 (27.1)	6 (22.2)	10 (31.2)		10 (30.3)	6 (23.1)	
Tumor stage[c], *N* (%)				0.002			0.003
I	3 (5.1)	2 (7.4)	1 (3.1)		2 (6.1)	1 (3.8)	
II	22 (37.3)	16 (59.3)	6 (18.8)		18 (54.5)	4 (15.4)	
III	32 (54.2)	9 (33.3)	23 (71.9)		13 (39.4)	19 (73.1)	
IV	2 (3.4)	0 (0.0)	2 (6.2)		0 (0.0)	2 (7.7)	

[a]S.E.M., standard error of mean.

[b]Individuals who smoked an average of <1 cigarette/day and for <1 year in their lifetime were defined as nonsmokers; otherwise, they were defined as smokers. Individuals were classified as drinkers if they drank at least twice a week and continuously for at least 1 year during their lifetime; otherwise, they were defined as nondrinkers.

[c]Tumor staging were reviewed by at least 3 pathologists and defined according to the American Joint Committee on Cancer (AJCC) 7th edition.
[d]High is defined as IHC Score > 6 and low is defined as IHC Score ≤ 6.
[e]*P* value was calculated by two-sided Chi-square test.

Supplementary Table 6. Univariate and multivariate Cox regression analysis for overall survival in ESCC patients.

Variable	Univariate			Multivariate		
	HR	95% CI	*P* value	HR[a]	95% CI[a]	*P* value
Gender (male vs. female)	2.04	0.79-5.26	0.134	-	-	-
Age (≥62 y vs. < 62 y)	1.71	0.87-3.37	0.116	-	-	-
Smoking (ever vs. never)	1.62	0.78-3.39	0.194	-	-	-
Drinking (ever vs. never)	1.00	0.51-1.96	0.995	-	-	-
Family history (yes vs. no)	0.94	0.43-2.07	0.874	-	-	-
Tumor stage (III/IV vs. I/II)	2.40	1.17-4.94	0.014	-	-	-
Differentiation (moderate/poor vs. well)	1.08	0.49-2.38	0.844	-	-	-
NSUN2 protein level (high vs. low)	3.52	1.67-7.41	< 0.001	3.52	1.46-8.49	0.005
GRB2 protein level (high vs. low)	3.31	1.66-6.60	< 0.001	3.64	1.57-8.41	0.003

[a]The HR and 95% CI of NSUN2 protein and GRB2 protein were calculated with adjustments for gender, age, smoking status, drinking status, family history, tumor stage and tumor differentiation as covariates. HR, hazard ratio; CI, confidence interval.

Supplementary Table 7. Characteristics of ESCC individuals for RNA-BisSeq and RNA-Seq in this study.

Sample ID	Sex	Age, year	Smoking status[a]	Drinking status[a]	Family history	Tumor stage[b]	Differentiation	Treatment
1	Male	68	Smoker	Drinker	No	III	Moderate	Surgery Only
2	Female	63	Smoker	Nondrinker	No	II	Moderate	Surgery Only
3	Female	66	Nonsmoker	Nondrinker	No	II	Well	Surgery Only
4	Male	51	Smoker	Drinker	No	IV	Moderate	Surgery Only
5	Male	69	Smoker	Drinker	No	III	Moderate	Surgery Only
6	Male	56	Nonsmoker	Nondrinker	No	III	Moderate	Surgery Only
7	Male	51	Smoker	Drinker	No	III	Moderate	Surgery Only

[a]Individuals who smoked an average of <1 cigarette/day and for <1 year in their lifetime were defined as nonsmokers; otherwise, they were defined as smokers. Individuals were classified as drinkers if they drank at least twice a week and continuously for at least 1 year during their lifetime; otherwise, they were defined as nondrinkers.

[b]Tumor staging were reviewed by at least 3 pathologists and defined according to the American Joint Committee on Cancer (AJCC) 7th edition.

Supplementary Table 8. Primers used in this study.

For qRT-PCR	Primer sequence (5' → 3')
NSUN2-Forward	GAACTTGCCTGGCACACAAAT
NSUN2-Reverse	TGCTAACAGCTTCTTGACGACTA
NSUN6-Forward	CAGAATGCCTTATTGTTAGGGCT
NSUN6-Reverse	ACCATATCAAGTTTAACCGCCTT
E2F1-Forward	ACGTGACGTGTCAGGACCT
E2F1-Reverse	GATCGGGCCTTGTTTGCTCTT
TFAP2C-Forward	CTGTTGCTGCACGATCAGACA
TFAP2C-Reverse	CTCAGTGGGGTTCATTACGGC
SP1-Forward	TGGCAGCAGTACCAATGGC
SP1-Reverse	CCAGGTAGTCCTGTCAGAACTT
NRF1-Forward	AGGAACACGGAGTGACCCAA
NRF1-Reverse	TATGCTCGGTGTAAGTAGCCA
GRB2-Forward	ATTCCTGCGGGACATAGAACA
GRB2-Reverse	GGTGACATAATTGCGGGGAAAC
LIN28B-Forward	CATCTCCATGATAAACCGAGAGG
LIN28B-Reverse	GTTACCCGTATTGACTCAAGGC
YBX1-Forward	AAGGAGAAAAGGGTGCGGAG
YBX1-Reverse	CCTACGACGTGGATAGCGTC
Rluc-Forward	ATATTGAGCCAGTAGCGCGG
Rluc-Reverse	GCCAAACAAGCACCCCAATC
Fluc-Forward	GTGTCCGATTCAGTCATGCC
Fluc-Reverse	CCAGCAGGGCAGATTGAATC
β-*Actin*-Forward	ACAGAGCCTCGCCTTTGCCGAT
β-*Actin*-Reverse	CTTGCACATGCCGGAGCCGTT
**For *Nsun2* knockout mice identification**	**Primer sequence (5' → 3')**
Nsun2-Forward	ACTGCCTACTACTCATGCCTTA
Nsun2-Reverse	GACAGCCTGGTCCTACACTC
**For m^5C-RIP-qPCR**	**Primer sequence (5' → 3')**
GRB2-Forward	CACATCCCACTGGATCTGGC
GRB2-Reverse	ATGACTTCCTCCTCCGCTCT
HRAS-Forward	CGCCCGCAACCCGAG
HRAS-Reverse	ACCGTTCACAGGCGCGA
PIK3R3-Forward	CCAGTTGCCACATGACTTGC
PIK3R3-Reverse	TCTCCCCCTCTACACACCAG
FGFR4-Forward	CTGACACAGTGCTCGACCTT
FGFR4-Reverse	AACCCTGACATTTGGGCCAT
CREB1-Forward	ATTTCTCATTTGGAGCCG
CREB1-Reverse	TGAACTCTTGACCACAGG
RASD2-Forward	ACCTGGCTCAGCAGGAG
RASD2-Reverse	AGGCGCAAGGGGCAGGT
PLCG1-Forward	TGGTGACCTCAGTCCCTTCA
PLCG1-Reverse	TAGCGGGATTCAAAGGAGCC
ITGAX-Forward	CAGGCTGCAGTATTTTGGGC

ITGAX-Reverse	CAGGTCCACCAGTCCATCCT
**For PAR-CLIP-qPCR**	**Primer sequence (5' → 3')**
GRB2-Forward	CACATCCCACTGGATCTGGC
GRB2-Reverse	ATGACTTCCTCCTCCGCTCT
**For ChIP-qPCR**	**Primer sequence (5' → 3')**
NSUN2-ChIP-Forward	GCCGTACACTGAGTTCGTC
NSUN2-ChIP-Reverse	GGAGGAGCGCCTGCTG

Supplementary Table 9. Antibodies utilized in this study.

Antibody	Application	Source	Catalog number
Rabbit anti-NSUN2	WB/IHC	Proteintech	20854-1-AP
Rabbit anti-GRB2	WB/IHC	Abcam	ab32037
Mouse anti-E2F1	WB/ChIP	Proteintech	66515-1-Ig
Rabbit anti-LIN28B	WB/IP	Proteintech	24017-1-AP
Rabbit anti-HSP90B1	WB	Proteintech	14700-1-AP
Rabbit anti-PKM	WB	Proteintech	10078-2-AP
Rabbit anti-CSDE1	WB	Proteintech	13319-1-AP
Rabbit anti-ENO1	WB	Proteintech	11204-1-AP
Rabbit anti-CDC5L	WB	Proteintech	12974-1-AP
Rabbit anti-SPIRE1	WB	Signalway Antibody	47711
Rabbit anti-AKT1/2/3	WB	Abcam	ab126811
Rabbit anti-phospho AKT	WB	Cell Signaling Technology	4060S
Rabbit anti-ERK1/2	WB	Abcam	ab17942
Rabbit anti-phospho ERK1/2	WB	Abcam	ab76299
Rabbit anti-MEK1/2	WB	Abcam	ab178876
Rabbit anti-phospho MEK1/2	WB	Abcam	ab194754
Mouse anti-5-methylcytosine	IP	Abcam	ab10805
Mouse anti-FLAG	WB	Sigma-Aldrich	F1804
Mouse anti-ACTIN	WB	Proteintech	66009-1-Ig

Supplementary Table 10. Targeted sequences of shRNAs or siRNAs and probe sequences of *GRB2* used in this study.

**Targeted sequences of shRNA or siRNA (5' → 3')**	
sh*NSUN2*-#1	CACGTGTTCACTAAACCCTAT
sh*NSUN2*-#2	GCTTGCTGATGTGTCTAAT
sh*GRB2*-#1	CCCAAGAACTACATAGAAA
sh*GRB2*-#2	CCAGAAACCAGCAGATATT
sh*LIN28B*-#1	GGATATTCCAGTCGATGTATT
sh*LIN28B*-#2	GCCATTACTGTCAGAGCATCA
si*E2F1*-#1	GCATCCAGCTCATTGCCAA
si*E2F1*-#2	CCTCTTCGACTGTGACTTT
si*SP1*-#1	GCGTTTCTGCAGCTACCTT
si*SP1*-#2	CCATTAACCTCAGTGCATT
si*TFAP2C*-#1	GCACGATCAGACAGTCATT
si*TFAP2C*-#2	CCAGTGGCAGAATATTTAA
si*NRF1*-#1	GCCACAGCCACACATAGTA
si*NRF1*-#2	GGAAACTTCGAGCCACGTT
si*YBX1*-#1	GGATATGGTTTCATCAACA
si*YBX1*-#2	CGTAACCATTATAGACGCT
**Probe sequences for RNA pulldown and REMSA (5' → 3')**	
GRB2 [C]	GGTCGGAAGCCTGTCCTCACCGTCTCGGGGGTTGTGGCCCCCGCCCCCTC-biotin
GRB2 [m^5C]	GGTCGGAAGCCTGTCCTCACCGTCT[m^5C]GGGGGTTGTGGCCCCCGCCCCCTC-biotin
